# In‐Silico Exploration of the StreptomeDB Database for Potential Irreversible DprE1 Inhibitors toward Antitubercular Treatment

**DOI:** 10.1002/open.202500237

**Published:** 2025-07-14

**Authors:** Doaa G. M. Mahmoud, Gamal A. H. Mekhemer, Mohamed‐Elamir F. Hegazy, Jabir H. Al‐Fahemi, Mahmoud A. A. Ibrahim

**Affiliations:** ^1^ Computational Chemistry Laboratory Chemistry Department Faculty of Science Minia University Minia 61519 Egypt; ^2^ Chemistry of Medicinal Plants Department National Research Centre 33 El‐Bohouth St. Dokki Giza 12622 Egypt; ^3^ Department of Chemistry Faculty of Science Umm Al‐Qura University Makkah 21955 Saudi Arabia; ^4^ Department of Engineering College of Engineering and Technology University of Technology and Applied Sciences Nizwa 611 Sultanate of Oman; ^5^ School of Health Sciences University of KwaZulu‐Natal Westville Campus Durban 4000 South Africa

**Keywords:** DprE1, irreversible covalent docking, MD simulations, streptomeDB, tuberculosis

## Abstract

Tuberculosis (TB) is one of the most fatal infectious diseases. Decaprenylphosphoryl‐D‐ribose oxidase (DprE1), one of the key enzymes in the synthesis of arabinogalactan and lipoarabinomannan, has become a focal point for anti‐TB drug discovery. An investigation of the StreptomeDB database, an extensive collection of natural products from Streptomyces species, yielded 63 nitro‐containing compounds with strong potential as masked electrophiles for covalent inhibitors. The compounds are prepared and screened against DprE1. The reliability of AutoDock 4.2.6 software in predicting the covalent docking scores and poses of the DprE1 inhibitors is evaluated. StreptomeDB compounds exhibiting covalent docking scores lower than PBTZ169, the reference inhibitor, against DprE1 (calc. –7.8 kcal mol^−1^) are recognized and underwent molecular dynamics simulations, succeeded by estimations of MM‐GBSA binding energies. According to the MM‐GBSA results obtained after 300 ns MDS, hydroxythaxtomin A and lajollamycin B exhibited better binding affinities against DprE1 with $\Delta G_{\text{binding}}$ values of –51.2 and –50.5 kcal mol^−1^, respectively, compared to PBTZ169 (calc. –49.3 kcal mol^−1^). Post‐MD analyses are conducted to examine the stability and affinity of the identified StreptomeDB compounds with DprE1. Robust bioavailability and drug‐likeness characteristics are expected for the investigated StreptomeDB compounds. These findings unveiled promising inhibitory activity for hydroxythaxtomin A and lajollamycin B against DprE1.

## Graphical abstract

StreptomeDB‐derived nitro compounds are screened against *Mycobacterium tuberculosis* decaprenylphosphoryl‐D‐riboseoxidase (DprE1) . Covalent docking, molecular dynamics simulations, and molecularmechanics/generalized Born surface area analyses identify hydroxythaxtomin and lajollamycin B as potent DprE1 inhibitors, surpassing the reference PBTZ169 in binding affinity. Drug‐likeness and bioavailability profiles support their potential as antituberculosis candidates.

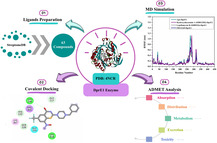

## 1 Introduction

Tuberculosis (TB), an infectious disease caused by *Mycobacterium tuberculosis*, constitutes a major and re‐emerging public health challenge globally with a high rate of incidence and mortality.^[^ ^1^ ^]^ The effective control of TB has reached a critical juncture due to the interplay between HIV infection together with the rise of multidrug‐resistant (MDR‐TB) and extensively drug‐resistant (XDR‐TB) strains of *M. tuberculosis*.^[^ ^2^, ^3^ ^]^ Genetic mutations in *M. tuberculosis* lead to resistance against widely utilized antibiotics, complicating TB treatment efficacy.^[^ ^4^, ^5^ ^]^ Furthermore, the COVID‐19 pandemic has caused a notable decrease in the recognition and documentation of TB cases.^[^ ^6^ ^]^ In 2020, an estimated 10.0 million individuals were infected with active TB, leading to 1.5 million deaths, underscoring the continued global burden of TB.^[^ ^7^ ^]^ Over the past years, the World Health Organization (WHO) has established ambitious targets to eradicate TB, striving for a remarkable 90% decrease in TB fatalities and an 80% decrease in incidence by the year 2030.^[^ ^8^ ^]^ Consequently, the urgent requirement for advanced and efficacious anti‐TB drugs is widely recognized. In this context, decaprenylphosphoryl‐D‐ribose oxidase (DprE1), an essential enzyme involved in the biosynthesis of the mycobacterial cell wall, has been identified as a promising target for the development of new antitubercular agents.^[^ ^9^, ^10^ ^]^ By targeting DprE1, it is possible to disrupt essential cellular processes and impede *M. tuberculosis*'s survival, thus offering a promising avenue for tackling drug resistance.^[^ ^10^, ^11^ ^]^ Various screening approaches were conducted to discover promising DprE1 inhibitors, resulting in the identification of both covalent and non‐covalent inhibitors.^[^ ^12^, ^13^ ^]^ Currently, four DprE1 inhibitors, including BTZ043, PBTZ169, TBA7371, and OPC167832, are advanced into clinical trials.^[^ ^14^, ^15^, ^16^, ^17^ ^]^ Notably, benzothiazinone derivatives (BTZs), particularly PBTZ169 (macozinone), have demonstrated superior potency in inhibiting DprE1.^[^ ^18^, ^19^ ^]^ Remarkably, BTZs are classified as mechanism‐based inhibitors that cause irreversible inactivation of DprE1.^[^ ^20^, ^21^ ^]^ Nitro‐containing compounds exhibit properties suitable for use as masked electrophiles in covalent inhibition of binding sites featuring cysteine and general acid residues.^[^ ^22^ ^]^ The critical factor in this action mechanism is an electron‐deficient warhead, which is a highly electrophilic chemical group facilitating covalent bonding with target proteins.^[^ ^23^, ^24^ ^]^ Specifically, the nitro group serves as such a warhead, enabling irreversible covalent bond formation with CYS387 in DprE1.^[^ ^21^, ^25^ ^]^ Based on a prior investigation, PBTZ169 offers several advantages over BTZ043, including low cost, an easy chemical synthesizing route, and an improved pharmacodynamics profile.^[^ ^26^ ^]^ Furthermore, preclinical studies revealed its synergistic effects when combined with bedaquiline and clofazimine.^[^ ^27^ ^]^

StreptomeDB is a comprehensive database that compiles a significant collection of natural products sourced from Streptomyces species, making it a valuable resource for the identification of novel drug candidates.^[^ ^28^ ^]^ Focused on the systematic documentation of these microbial metabolites, StreptomeDB plays an essential role in advancing drug discovery efforts by providing detailed insights into the biosynthetic pathways and pharmacological potential of the compounds.^[^ ^28^ ^]^

**Figure 1:**
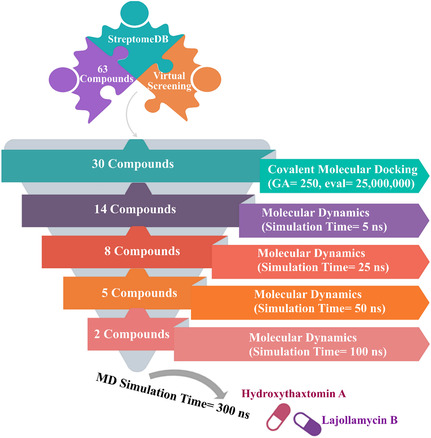
Flowchart diagram of the utilized in‐silico methodologies in filtering the StreptomeDB compounds.

Consequently, the current study aimed to identify promising DprE1 inhibitors, leveraging the StreptomeDB database. In this context, 63 nitro‐containing compounds sourced from the StreptomeDB database were evaluated through covalent docking simulations. Molecular dynamics simulations (MDS) for potent StreptomeDB compounds complexed with DprE1 were conducted. Binding energies (Δ*G*_binding_) for the StreptomeDB‐DprE1 complexes were evaluated during the MDS employing the MM‐GBSA technique. Post‐MD analyses were also conducted to assess the stability of the investigated StreptomeDB compounds in complex with DprE1 over the MDSs. The pharmacokinetic profile and drug‐likeness characteristics of the identified potent StreptomeDB compounds were predicted. **Figure** **1** illustrates the in‐silico workflow employed to screen the examined StreptomeDB compounds against DprE1. In this regard, the present work demonstrates that StreptomeDB compounds are potential anti‐TB inhibitors, requiring more in‐vivo/in‐vitro investigations.

## 2 Computational Methods

### 2.1 DprE1 Preparation

Gene–gene and protein–protein interaction networks (Figure S1, Supporting Information) observations underscore the central importance of DprE1 in the metabolic network of *M. tuberculosis*, providing additional support for its potential as a promising drug target. The 3D arrangement of *M. tuberculosis* DprE1, covalently linked to PBTZ169, was acquired from the Protein Data Bank database, featuring a resolution of 1.9 Å (PDB accession no. 4NCR).^[^ ^27^ ^]^ Using Modeller software, the non‐terminal missing residues were modeled to complete the DprE1 structure.^[^ ^29^ ^]^ To streamline the structure, all extraneous components, such as water molecules, heteroatoms, ions, and co‐crystallized ligand, were eliminated. Furthermore, polar hydrogens were incorporated into the structure at a pH of 7, employing the H++ web server.^[^ ^30^ ^]^

### 2.2 Covalent Ligands Preparation

In an effort to identify the most potent covalent inhibitors targeting DprE1, 63 nitro‐containing compounds were collected from the StreptomeDB database in SDF format.^[^ ^28^ ^]^ Employing the Omega2 software, 3D representations of each StreptomeDB compound were crafted.^[^ ^31^, ^32^ ^]^ Afterward, the generated structures underwent minimization employing the MMFF94S force field within the SZYBKI software.^[^ ^33^, ^34^ ^]^ Atomic charges for the StreptomeDB compounds were determined via the Gasteiger–Marsili method.^[^ ^35^ ^]^ Tautomer enumeration of the StreptomeDB compounds was carried out using the tautomer application implemented by means of the QUACPAC software package.^[^ ^36^ ^]^ Finally, the FixpKa program, functioning within QUACPAC, was utilized to predict the most probable molecular ionization status.

### 2.3 Covalent Docking Protocol

As a part of this in‐silico study, covalent docking calculations were conducted with the AutoDock4.2.6 software,^[^ ^37^ ^]^ leveraging the flexible side chain method.^[^ ^38^ ^]^ In this method, the ligand is overlapped with the protein residue, and subsequently used AutoDock to optimize its conformation.^[^ ^38^ ^]^ To comply with the AutoDock4.2.6 protocol, the pdb file of DprE1 was converted to a pdbqt file.^[^ ^39^ ^]^ Concerning the covalent docking parameters, the default values were maintained, apart from the number of genetic algorithm (*GA*) runs, which was set to 250, and the maximum number of energy evaluations (*eval*), which was adjusted to 25 000 000. To ensure accurate binding predictions, a grid box was defined to encompass the DprE1 binding site. The grid box with dimensions of 40 Å × 40 Å × 40 Å was carefully chosen, and centered at coordinates *x* = 17.176, *y* = −20.119, and *z* = 1.875. A grid spacing of 0.375 Å was employed.

### 2.4 Molecular Dynamics Simulations (MDS)

The most promising StreptomeDB compounds in complex with DprE1 underwent MDS to evaluate their long‐term stability using AMBER20 software.^[^ ^40^ ^]^ Technical details regarding the employed MDSs can be found in Ref. [41, 42, 43, 44, 45] Succinctly, the GAFF2 (General AMBER force field) was applied to describe the investigated StreptomeDB compounds.^[^ ^46^ ^]^ However, DprE1 was described by relying on the FF14SB AMBER force field.^[^ ^47^ ^]^ To facilitate atomic charge estimates, N‐terminal acetyl, and C‐terminal methyl amide groups were added to cap the irreversible covalent inhibitors with CYS387 residue. Subsequently, geometric optimization was performed using the Gaussian09 software.^[^ ^48^ ^]^ The B3LYP/6‐31 G* level of theory was employed for optimization. The atomic charges of the StreptomeDB compounds were then computed employing the restrained electrostatic potential (RESP) fitting technique at the HF/6‐31 G* level.^[^ ^49^ ^]^ An antechamber module, an integral component of the AMBER package, was applied to define the parameters and the atom types of covalent inhibitors with the CYS387 residue. Following that, the ligand‐DprE1 complexes were dissolved in a truncated octahedron box filled with TIP3P water molecules, maintaining an average distance of 12 Å from the solute edge. To neutralize the solvated complexes, the appropriate quantity of Na^+^/Cl^−^ ions was added. Moreover, 0.15 M NaCl was incorporated to preserve physiological ionic conditions. The solvated complexes then underwent energy minimization for 5000 cycles. After minimization, the ligand‐DprE1 complexes were heated incrementally to 310 K over 50 ps, and then an equilibration simulation of 10 ns was performed under NPT conditions. Subsequently, production MDS of the equilibrated complexes were run over 5, 25, 50, 100, and 300 ns, recording snapshots at intervals of 10 ps. Bonds involving hydrogens were *restricted* utilizing the SHAKE algorithm with a time step of 2 fs.^[^ ^50^ ^]^ Additionally, the long‐range electrostatic forces were handled utilizing the particle mesh Ewald (PME) method.^[^ ^51^ ^]^ Lennard‐Jones interactions were assessed with a 12 Å cutoff. Throughout the production steps, an NTP ensemble was sustained with a Langevin thermostat (310 K) and an anisotropic Berendsen barostat (1 atm).^[^ ^52^, ^53^ ^]^ The PMEMD.CUDA GPU, as implemented in the AMBER20 software, was utilized to carry out MDS. Ultimately, graphical representations of the investigated StreptomeDB compounds were generated with the help of the BIOVIA Materials Studio software.^[^ ^54^ ^]^

### 2.5 MM‐GBSA Binding Affinity

For determining the binding energies (Δ*G*_binding_) of the top StreptomeDB compounds complexed with DprE1, a molecular mechanics‐generalized Born surface area (MM‐GBSA) approach was implemented.^[^ ^55^ ^]^ Due to its lower computational cost and time, the MM‐GBSA approach was selected for binding energy calculations over other approaches, such as molecular mechanics‐Poisson Boltzmann surface area (MM‐PBSA).^[^ ^56^ ^]^ The energy contribution from polar solvation was evaluated using the generalized Born (GB) model (igb = 2) proposed by Onufriev et al..^[^ ^57^ ^]^ The corresponding binding energies (Δ*G*_binding_) were estimated relying on the uncorrelated snapshots compiled from MDS, using the following equation

(1) $$\Delta G_{\text{binding}}=G_{\text{Complex}}-\left(G_{\text{DprE}1\;}+G_{\text{StreptomeDB compound }}\right)$$

In which the *G* expression is

(2) $$G=E_{\text{MM}}+G_{\text{solv}}-TS$$

(3) $$E_{\text{MM}}=E_{\text{vdw}}+E_{\text{ele}}+E_{\text{int}}$$

(4) $$E_{\text{int}}=E_{\text{bond}}+E_{\text{angle}}+E_{\text{torsion}}$$

*E*_MM_ stands for the energy of molecular mechanics (MM) in the gas phase. *G*_solv_ denotes the solvation energy. *E*_int_ signifies the internal MM energy, involving bond (*E*_bond_), angle (*E*_angle_), and dihedral (*E*_torsion_) energies. Electrostatic interactions and van der Waals forces are represented by *E*_ele_ and *E*_vdW_, respectively. A single‐trajectory technique was employed to obtain the spatial coordinates for the covalent inhibitors, DprE1, and the ligand‐DprE1 complexes. For all examined StreptomeDB compounds, the consideration of entropy contribution (*S*) was avoided owing to its expensive computational cost.^[^ ^58^, ^59^ ^]^

### 2.6 Physicochemical Features

The molecular characteristics have a crucial role to play in shaping the effectiveness of therapeutic medications, highlighting their profound impact on treatment outcomes.^[^ ^60^ ^]^ The drug‐likeness features were evaluated utilizing an online SWISS‐ADME package (http://www.swissadme.ch). For each investigated StreptomeDB compound, the following molecular properties were estimated: molecular weight (MW); H‐bond donors (HBD); Mlog*P* (*n*‐octanol/water partition coefficient); H‐bond acceptors (HBA); and topological polar surface area (TPSA).

### 2.7 ADMET

Evaluating the ADMET characteristics, including chemical absorption, distribution, metabolism, excretion, and toxicity, is pivotal in ascertaining the therapeutic efficacy of prospective drug candidates.^[^ ^61^ ^]^ To expedite the evaluation process and minimize the risk of drug failure, the pkCSM server (http://biosig.unimelb.edu.au/pkcsm/prediction) was employed for the prediction of ADMET features for the identified StreptomeDB compounds. The absorption (A) property was assessed by anticipating the Caco‐2 permeability and skin permeability (log *Kp*). The distribution (D) characteristic was evaluated by investigating the values of the logarithm of the log BB (blood‐brain barrier) partition coefficient and CNS (central nervous system) permeability. Using cytochromes P450 (CYPs) models, predictions regarding metabolism (M) were conducted. Estimation of the excretion (E) property was made using the total clearance of the inhibitor. Furthermore, the toxicity (T) was examined by considering parameters such as AMES toxicity, maximum tolerated dose (MTD), hERG inhibition, and skin sensitization.

## 3 Results and Discussion

### 3.1 Covalent Docking Assessment

**Figure 2:**
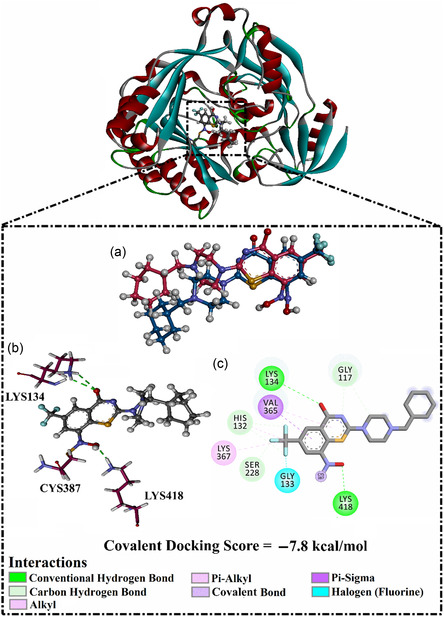
a) Superimposed of the predicted (dark cyan) and experimental (dark pink) binding modes, and b) 3D and c) 2D depictions of the interactions of PBTZ169 with DprE1.

In an effort to ensure the reliability of the employed covalent docking protocol using AutoDock4.2.6 software, a validation process was conducted. First, the co‐crystalized PBTZ169 (macozinone) inhibitor was prepared and re‐docked against the DprE1. The RMSD between the co‐crystalized and the predicted binding modes was estimated and found to be 0.73 Å (**Figure** **2**).^[^ ^62^, ^63^ ^]^

Furthermore, the covalent docking computations demonstrated a favorable docking score of −7.8 kcal mol^−1^ for PBTZ169 complexed with DprE1. The strong docking score likely results from the formation of an irreversible covalent bond between the nitro group and the sulfur atom of CYS387 (1.16 Å), accompanied by the establishment of two H‐bonds (Figure 2). More exactly, an H‐bond formed between the oxygen atom of the thiazine‐4‐one and the NH_3_ group of LYS134 (2.52 Å). In addition, the NO_2_ group established an H‐bond with the NH_3_ group of LYS418 (2.00 Å). Besides hydrogen bonding, an array of other interactions was detected, including C–H bonds with HIS132, GLY117, and SER228 residues (Figure 2). As well, interactions such as halogen, π‐sigma, alkyl, and π‐alkyl were found between PBTZ169 and GLY133, VAL365, LYS367, and HIS132 residues, respectively (Figure 2). These insightful findings highlight the exceptional proficiency of AutoDock4.2.6 in accurately forecasting the ligand‐DprE1 binding mode, solidifying its application in the subsequent calculations.

### 3.2 Screening of StreptomeDB Database

At the outset of drug discovery, virtual screening emerges as a powerful technique for rapidly identifying potential bioactive inhibitors.^[^ ^64^, ^65^ ^]^ To this end, an extensive covalent docking computation was conducted to screen a set of 63 nitro‐containing compounds sourced from the StreptomeDB database against DprE1. The covalent docking study was performed with specific parameters. Specifically, the *GA* value was adjusted to 250, and the *eval* value was fixed at 25 000 000. Presented in Table S1, Supporting Information are the computed covalent docking scores for the investigated StreptomeDB compounds. Relying on the covalent docking scores, just 30 StreptomeDB compounds demonstrated docking scores lower than the calculated score of PBTZ169 (−7.8 kcal mol^−1^). Depicted in Figure S2, Supporting Information are the molecular interactions of the potent compounds complexed with DprE1, represented in both 2D and 3D formats. Valuable insights into the 2D chemical structures, estimated covalent docking scores, and binding features of the most promising StreptomeDB compounds with DprE1 can be found in **Table** **1**. It is pertinent to mention that the hydroxythaxtomin A (SDB12202) and lajollamycin B (SDB9226) were chosen relying on the estimated MM‐GBSA binding energy throughout a 300 ns MDS, as will be elaborated in the following sections.

**Table 1:** Computed covalent docking scores (in kcal mol^−1^), 2D chemical structures, and binding features of PBTZ169 and the most promising StreptomeDB compounds inside the DprE1 binding pocket.

No.	StreptomeDB Code/Namea)	2D Chemical Structure	Covalent Docking Score [kcal mol^−1^]	Binding Featuresb)
	PBTZ169 (Macozinone)	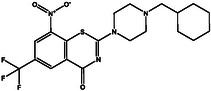	− 7.8	LYS134 (2.52 Å), LYS418 (2.00 Å)
**1**	SDB12202 (Hydroxythaxtomin A)	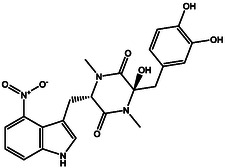	− 14.2	HIS132 (2.19 Å), ASP318 (2.33 Å), GLN336 (2.16 Å), TYR415 (2.09, 2.20 Å)
**2**	SDB9226 (Lajollamycin B)	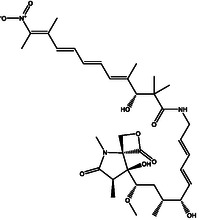	− 13.9	ARG58 (2.00 Å), HIS132 (1.87 Å), TYR327 (2.80 Å), ALA330 (2.11 Å), GLN336 (2.32 Å), LYS418 (1.83 Å)

a) The potent StreptomeDB compounds ranked relying on the covalent docking score.

b) Only conventional H‐bonds (in Å) are presented.

Upon analyzing the data presented in Table 1 and Figure S2, Supporting Information, it was observed that a significant proportion of the investigated StreptomeDB compounds exhibited remarkably similar docking poses within the DprE1 binding pocket. These identified StreptomeDB compounds demonstrated the ability to form an irreversible covalent bond with CYS387 and many hydrogen bonds with key residues such as HIS132, GLN336, and LYS418. Furthermore, π‐based and hydrophobic interactions were prominently indicated between the investigated StreptomeDB compounds and key amino acids within the DprE1 binding site.

**Figure 3:**
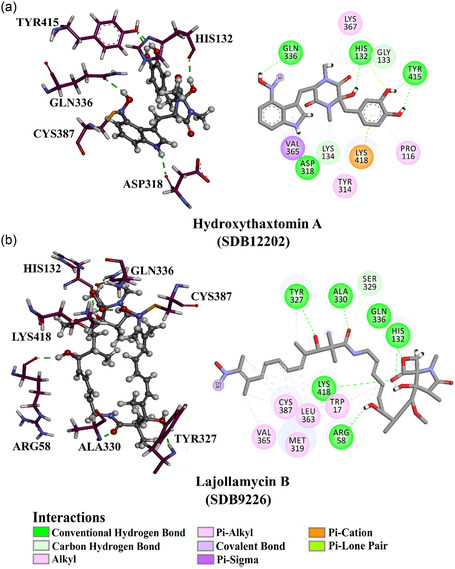
2D and 3D molecular interactions of a) hydroxythaxtomin A and b) lajollamycin B complexed with DprE1.

To enhance visual comprehension, **Figure** **3** provides comprehensive 2D and 3D representations of hydroxythaxtomin A and lajollamycin B; the nitro (NO_2_) group of the two inhibitors created an irreversible covalent bond with the sulfur atom of CYS387 (1.75 and 1.32 Å, respectively) (Figure 3).

As outlined in Table 1, hydroxythaxtomin A, a herbicide with favorable toxicological properties,^[^ ^66^ ^]^ exhibited the most favorable covalent docking score of −14.2 kcal mol^−1^ toward DprE1. This promising docking score of hydroxythaxtomin A toward DprE1 can be ascribed to the creation of five intermolecular H‐bonds with the essential residues of DprE1 (Figure 3). Precisely, the OH group of hydroxy‐piperazine‐dione exhibited an H‐bond with the C=O group of HIS132 (2.19 Å). Also, the NH group of the pyrrole established an H‐bond with the backbone C=O of ASP318 (2.33 Å). The oxygen of the nitro group formed an H‐bond with the C=O group of GLN336 (2.16 Å). Moreover, the two OH groups of pyrocatechol contributed to two H‐bonds with the OH group of TYR415 (2.09, 2.20 Å). These interactions indicate a dual inhibition mechanism. Covalent inactivation occurs through irreversible binding to CYS387, while structural destabilization results from non‐covalent interactions with HIS132, ASP318, GLN336, and TYR415. Modification of CYS387 likely disrupts redox chemistry essential for epimerization. HIS132 and TYR415 are crucial for substrate binding, and stable hydrogen bonds with them may obstruct substrate access or proton relay. Additionally, ASP318 and GLN336 may help stabilize an inactive enzyme form. Thus, hydroxythaxtomin A may function as a competitive or mechanism‐based inhibitor of DprE1, affecting mycobacterial cell wall biosynthesis.

Lajollamycin B demonstrates potent anti‐cancer properties and significant antimicrobial activity, making it a promising candidate for combatting cancer and microbial infections.^[^ ^67^, ^68^ ^]^ Lajollamycin B exhibited the second superior covalent docking score with DprE1, recording a value of –13.9 kcal mol^−1^. In addition, as outlined in Table 1, lajollamycin B participated in the formation of six H‐bonds with key amino acid residues located within the DprE1 binding pocket. In detail, the OH group (i.e., *N*‐(*2 E,4 E,6 R,7S,9 R*)‐6‐hydroxy) exhibited an H‐bond with the C=O group of ARG58 (2.00 Å) (Figure 3). The OH group of hydroxypyrrolidinone demonstrated an H‐bond with the C=O group of HIS132 (1.87 Å). Besides, the OH group (i.e., (*R,4Z,6 E,8 E,10Z*)‐3‐hydroxy) contributed to an H‐bond with the NH group of TYR327 (2.80 Å). An H‐bond was anticipated between the C=O group of 11‐nitrododeca‐4,6,8,10 tetraenamide and the NH group of ALA330 (2.11 Å) (Figure 3). The oxygen atom and the C=O group of oxetanone exhibited two H‐bonds with the NH_2_ group of the GLN336 (2.32 Å) and the NH_3_ group of LYS418 (1.83 Å), respectively. These interactions indicate a potential inhibitory mechanism where lajollamycin B may hinder DprE1's catalytic function by targeting residues essential for enzymatic activity and stability. Covalent bonding with the nucleophilic CYS387 likely leads to irreversible inactivation. The interaction with HIS132 may obstruct substrate positioning or proton transfer, while GLN336 and LYS418 could limit the conformational flexibility necessary for enzyme activity. Additionally, residues like ARG58, TYR327, and ALA330 may help stabilize an inactive enzyme state. Collectively, these interactions suggest that lajollamycin B irreversibly alkylates the active site cysteine and promotes an inactive conformation, effectively inhibiting DprE1 and disrupting mycobacterial cell wall synthesis.

Overall, hydroxythaxtomin A and lajollamycin B exhibited lower docking scores with DprE1 than PBTZ169 (calc. −7.8 kcal mol^−1^), suggesting that both investigated compounds have the potential to be DprE1 inhibitors.

### 3.3 MDS

**Figure 4:**
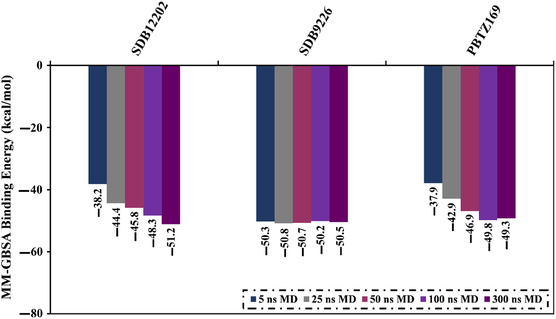
Evaluated binding energies of the most potent StreptomeDB compounds and PBTZ169 toward DprE1 over 300 ns MDS.

MDS serves as an effective strategy to validate detailed findings related to protein–ligand interactions with a dynamic aspect.^[^ ^69^, ^70^ ^]^ Herein, MDS was performed on the 30 potent StreptomeDB compounds complexed with DprE1, which were selected based on estimated covalent docking scores less than the calculated score of PBTZ169 (–7.8 kcal mol^−1^). To streamline computational resources and minimize time requirements, a short MDS of 5 ns was performed, succeeded by calculations of MM‐GBSA binding energies (Table S2, Supporting Information). As implied in Table S2, Supporting Information, only 14 StreptomeDB compounds out of the 30 StreptomeDB compounds had Δ*G*_binding_ lower than that of PBTZ169 (calc. –37.9 kcal mol^−1^) with DprE1. To ensure reliable outcomes, these 14 StreptomeDB compounds complexed with DprE1 underwent 25 ns MDS, followed by an estimation of the corresponding binding energies (Table S3, Supporting Information). Notably, Table S3, Supporting Information indicated that 8 compounds demonstrated Δ*G*_binding_ less than that of PBTZ169 (calc. –42.9 kcal mol^−1^), which served as the threshold for identifying potent DprE1 inhibitors. These 8 potent compounds complexed with DprE1 underwent MDS for a longer time of 50 ns, and the corresponding binding energies were compiled and gathered in Table S4, Supporting Information. As enrolled in Table S4, Supporting Information, 5 out of 8 StreptomeDB compounds demonstrated binding energies less than −45.1 kcal mol^−1^. The MDSs of these StreptomeDB compounds complexed with DprE1 were prolonged to 100 ns. Moreover, the corresponding binding affinities were computed (Table S5, Supporting Information). Intriguingly, from this set of 5 compounds, the binding affinities calculations indicated that lajollamycin B (SDB9226) and hydroxythaxtomin A (SDB12202) exhibited significant binding energies (Δ*G*_binding_) of −50.2 and −48.3 kcal mol^−1^, respectively, compared to PBTZ169 (calc. –49.8 kcal mol^−1^) (Table S5, Supporting Information). To further enhance the precision of binding energies, the MDS for the 2 investigated StreptomeDB compounds was elongated to 300 ns. Additionally, the binding energies of these identified StreptomeDB compounds with DprE1 over 300 ns MDS were calculated and displayed in **Figure** **4**. Compared to PBTZ169 (calc. −49.3 kcal mol^−1^), the estimated MM‐GBSA//300 ns MDS of hydroxythaxtomin A and lajollamycin B were −51.2 and −50.5 kcal mol^−1^, respectively. As per data portrayed in Figure 4, the Δ*G*_binding_ of lajollamycin B remained relatively unchanged throughout 100 and 300 ns MDS against DprE1. As indicated by employing long MDS, hydroxythaxtomin A and lajollamycin B are prospective DprE1 inhibitors and thus hold potential as antituberculosis drug candidates.

To examine the binding of hydroxythaxtomin A, lajollamycin B, and PBTZ169, the calculated binding affinities were decomposed into their individual components (**Table** **2**). According to the MM‐GBSA binding energy decomposition, hydroxythaxtomin A, lajollamycin B, and PBTZ169 in complex with DprE1 exhibited electrostatic (Δ*E*_ele_) interactions, with an average value of −86.9, −66.7, and −20.4 kcal mol^−1^, respectively. Furthermore, van der Waals (Δ*E*_vdW_) interactions were crucial in the binding of hydroxythaxtomin A, lajollamycin B, and PBTZ169, exhibiting average values of −47.2, −61.5, and −52.1 kcal mol^−1^, respectively. Looking at Table 2, hydroxythaxtomin A, lajollamycin B, and PBTZ169 complexed with DprE1 contribute modestly to Δ*E*_int_, with average values of 7.9, 3.2, and 5.3 kcal mol^−1^, respectively (Table 2).

**Table 2:** MM‐GBSA binding energy components of hydroxythaxtomin a, lajollamycin B, and co‐crystallized PBTZ169 inhibitor with DprE1 during the 300 ns MDS.

StreptomeDB Code/Name	Calculated MM‐GBSA binding energy [kcal mol^−1^]							
	Δ*E*_vdw_	Δ*E*_ele_	Δ*E*_int_	Δ*E*_GB_	Δ*E*_SUR_	Δ*G*_gas_	Δ*G*_Solv_	Δ*G*_binding_
PBTZ169 (Macozinone)	–52.1	–20.4	5.3	46.4	–6.5	–89.2	40.0	–49.3
(SDB12202) Hydroxythaxtomin A	–47.2	–86.9	7.9	78.8	–7.2	–122.8	71.6	–51.2
(SDB9226) Lajollamycin B	–61.5	–66.7	3.2	73.6	–8.6	–115.5	65.0	–50.5

**Figure 5:**
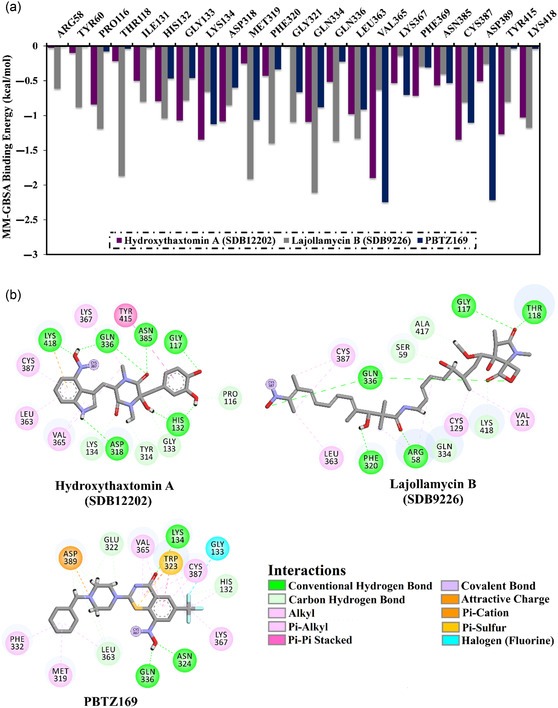
a) MM‐GBSA per‐residue decomposition energies and b) 2D molecular interactions of hydroxythaxtomin A, lajollamycin B, and PBTZ169 with DprE1 relying on the final trajectory through 300 ns MDS.

Toward deeper insight into their binding modes and intermolecular interaction, energy decomposition per‐residue was carried out for hydroxythaxtomin A, lajollamycin B, and PBTZ169 with DprE1. A specific focus was placed on amino acids that had energy contributions below −0.5 kcal mol^−1^ (**Figure** **5**a). Based on the data presented in Figure 5a, LYS134, GLN334, VAL365, and CYS387 residues conferred notable contributions to the binding of hydroxythaxtomin A, lajollamycin B, and PBTZ169 with DprE1. Notably, the investigated complexes displayed remarkably consistent interaction patterns with significant amino acid residues. CYS387 residue played a major role in the overall Δ*G*_binding_, contributing significantly with values of −1.3, −0.8, and −1.1 kcal mol^−1^ for hydroxythaxtomin A‐, lajollamycin B‐, and PBTZ169‐DprE1 complexes, respectively (Figure 5a).

Additionally, the 2D visualizations of the final snapshot of 300 ns MDS for hydroxythaxtomin A, lajollamycin B, and PBTZ169 with DprE1 are depicted in Figure 5b. Interestingly, those investigated StreptomeDB compounds in complex with DprE1 exhibited strikingly similar interaction patterns with nearby residues, suggesting similarity in their binding modes. Obviously, hydroxythaxtomin A retained the essential H‐bonds with HIS132, ASP318, and GLN336 and formed new H‐bonds with GLY117, ASN385, and LYS418. Moreover, lajollamycin B preserved the essential H‐bonds with ARG58 and GLN336 and exhibited new H‐bonds with GLY117, THR118, and PHE320. This highlights the importance of conducting MDS to get more reliable results.

### 3.4 Post‐MD Analyses

The steadiness of hydroxythaxtomin A (SDB12202) and lajollamycin B (SDB9226) complexed with DprE1 was assessed through structural and energetic analyses during 300 ns MDS. Post‐MD analyses included the binding energy per frame, CoM distance, RMSD, RMSF, Rg, and the number of H‐bonds.

#### 3.4.1 Binding Energy Per Frame

**Figure 6:**
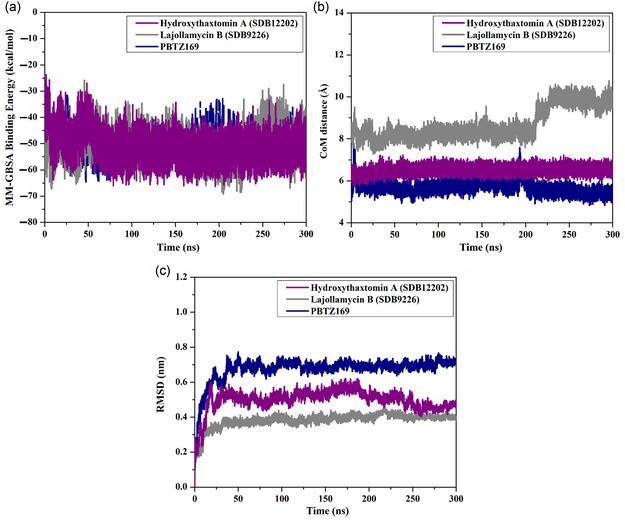
Estimated a) binding energy per frame, b) CoM distances, and c) RMSD of hydroxythaxtomin A (purple), lajollamycin B (gray), and PBTZ169 (dark blue) within DprE1 over 300 ns MDS.

The steadiness of hydroxythaxtomin A and lajollamycin B with DprE1 was examined throughout 300 ns, focusing on the correlation between binding energy and simulation time (**Figure** **6**a). According to data presented in Figure 6a, hydroxythaxtomin A and lajollamycin B demonstrated overall energetic stability over the course of 300 ns MDS with Δ*G*_binding_ values of –51.2 and –50.5, respectively, compared to –49.3 kcal mol^−1^ for PBTZ169. These intriguing findings underscored the enduring stability of the investigated StreptomeDB compounds with DprE1 throughout the entire MDS.

#### 3.4.2 Center‐of‐Mass Distance (CoM)

CoM distance between the examined StreptomeDB compounds and the CYS387 residue of DprE1 was meticulously examined and plotted in Figure 6b. The average CoM distances were 6.5, 8.7, and 5.7 Å for hydroxythaxtomin A‐, lajollamycin B‐, and PBTZ169‐DprE1 complexes, respectively. From the measured CoM results, it is evident that the inspected StreptomeDB compounds maintained a state of complete steadiness within the binding pocket of DprE1.

#### 3.4.3 Root‐Mean‐Square Deviation (RMSD)

The RMSD of backbone Cα atoms was conducted to track structural changes of the inspected StreptomeDB‐DprE1 complexes over 300 ns MDS (Figure 6c). In conformity with Figure 6c, the RMSD values for hydroxythaxtomin A‐, lajollamycin B‐, and PBTZ169‐DprE1 complexes were revealed to be 0.50, 0.38, and 0.67 nm, respectively. Based on these results, it is evident that the examined StreptomeDB compounds maintained a strong binding affinity to the DprE1 binding site without disrupting its overall structure.

To further evaluate the stability of the StreptomeDB‐DprE1 complexes, the relative RMSD of the ligands with respect to the DprE1 backbone was also calculated and presented in Figure S3, Supporting Information. Consistent with Figure S3, Supporting Information, the RMSD values for hydroxythaxtomin A, lajollamycin B, and PBTZ169 were found to be 0.09, 0.17, and 0.21 nm, respectively. Lower and stable RMSD values across the simulation indicated that the identified StreptomeDB compounds remained consistently bound within the active site without significant displacement.

#### 3.4.4 Root‐Mean‐Square Fluctuation (RMSF)

**Figure 7:**
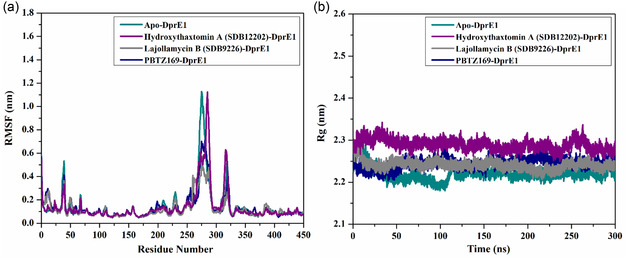
a) RMSF and b) Rg of apo‐DprE1 (cyan), hydroxythaxtomin A‐DprE1 (purple), lajollamycin B‐DprE1 (gray), and PBTZ169‐DprE1 (dark blue) complexes throughout 300 ns MDS.

The Cα atom is fundamental in defining the backbone direction of each amino acid within the DprE1 chain. **Figure** **7**a shows the RMSF values (in nm) for the Cα atoms, providing insights into the residue flexibility within the DprE1 structure. The apo‐, hydroxythaxtomin A‐, lajollamycin B‐, and PBTZ169‐DprE1 complexes demonstrated average RMSF values of 0.14, 0.14, 0.12, and 0.14 nm (Figure 7a). The RMSF analysis revealed significant fluctuations in certain DprE1 residues within the 260–290 and 310–330 ranges, implying that the interactions between the identified StreptomeDB compounds and DprE1 imposed various constraints on the motions near these amino acids (Figure 7a). The observed variations in RMSF values may reflect differences in the internal dynamics and interaction intensities.

#### 3.4.5 Radius of Gyration (Rg)

The compactness and stability of the apo‐ and the soaked‐DprE1 were assessed by monitoring the Rg during the 300 ns MDS (Figure 7b). As illustrated in Figure 7b, the average *Rg* values for apo‐, hydroxythaxtomin A‐, lajollamycin B‐, and PBTZ169‐DprE1 complexes were 2.22, 2.29, 2.24, and 2.24 nm, respectively. Notably, DprE1 maintained its compact conformation when bound to hydroxythaxtomin A, lajollamycin B, and PBTZ169 through the 300 ns MDS, as evidenced by the Rg data. According to the *R*g analysis, DprE1 preserved a compact conformational state upon binding with the identified StreptomeDB compounds throughout 300 ns MDS. These results strongly indicated that the binding of these drugs considerably enhanced the structural stability of DprE1.

#### 3.4.6 H‐Bond Analysis

**Figure 8:**
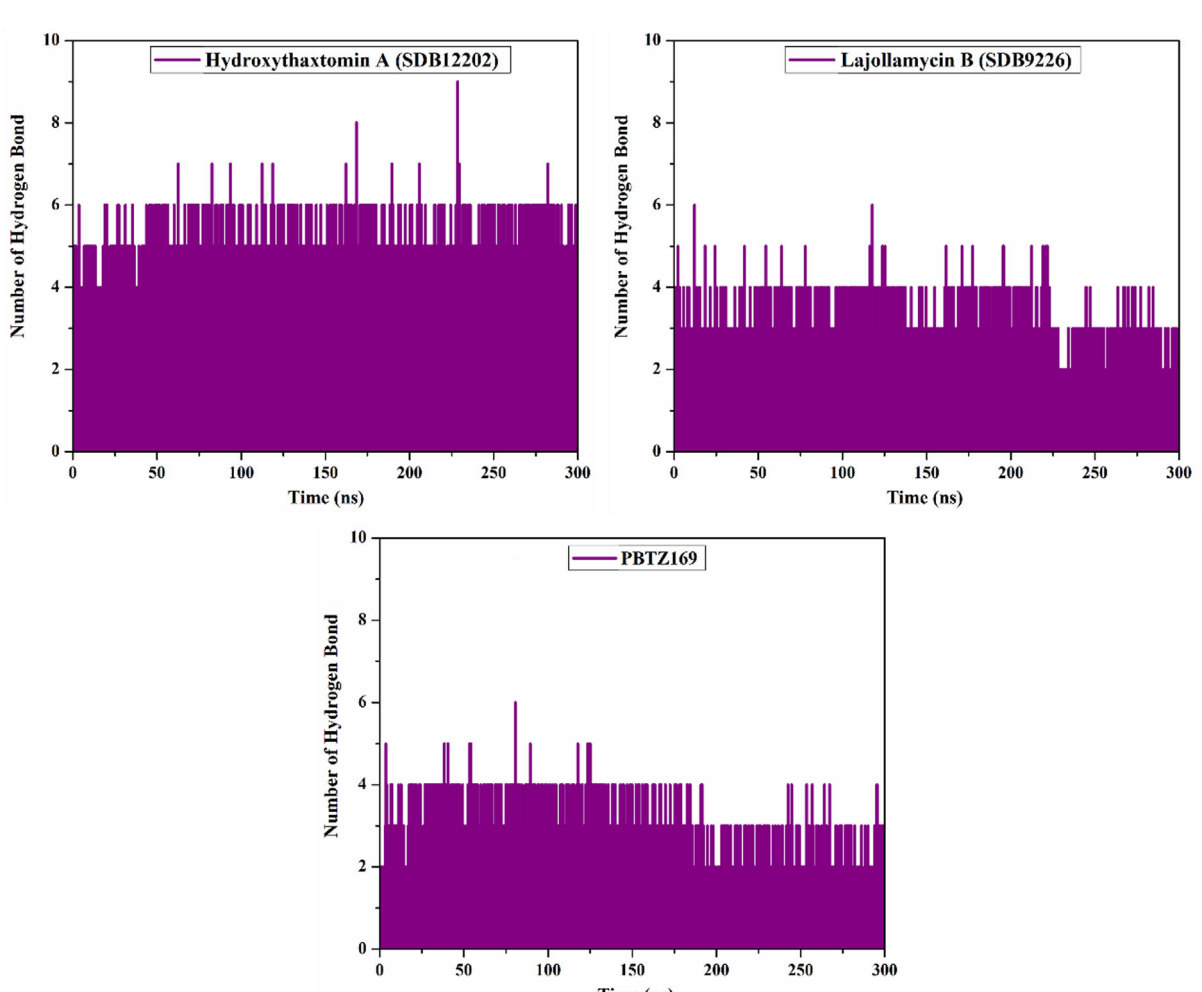
Number of H‐bonds of hydroxythaxtomin A, lajollamycin B, and PBTZ169 with DprE1 throughout 300 ns MDS.

The number of H‐bonds versus time was estimated to comprehend the stability of hydroxythaxtomin A, lajollamycin B, and PBTZ169 inside the DprE1 binding site over the course of 300 ns MDS (**Figure** **8**). Notably, the average number of H‐bonds observed for hydroxythaxtomin A, lajollamycin B, and PBTZ169 complexed with DprE1 were 5, 4, and 3, respectively. The H‐bond analysis demonstrated the steadiness of examined StreptomeDB compounds within the DprE1 binding site during the entire MDS.

### 3.5 Physicochemical Features

**Figure 9:**
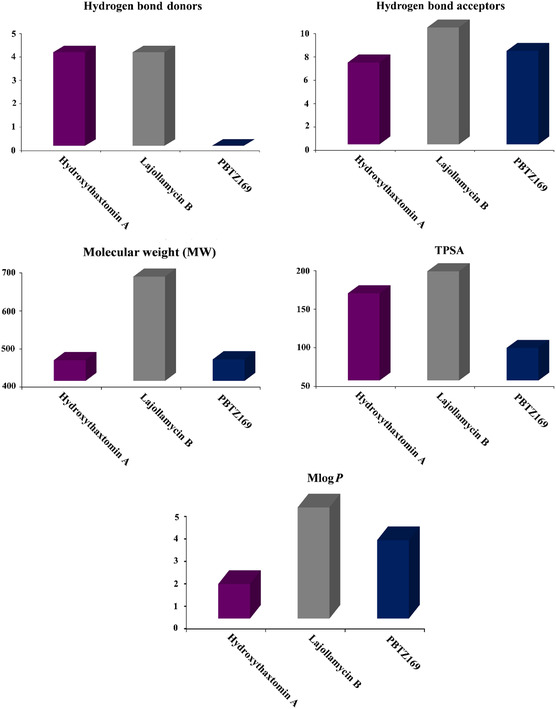
Predicted drug‐likeness characteristics of hydroxythaxtomin A, lajollamycin B, and PBTZ169 as DprE1 inhibitors.

The drug‐likeness properties and bioactivity of hydroxythaxtomin A (SDB12202) and lajollamycin B (SDB9226) as DprE1 inhibitors were assessed using SWISS‐ADME server,^[^ ^71^ ^]^ compared to those of PBTZ169 (**Figure** **9**). Notably, the HBD of hydroxythaxtomin A, lajollamycin B, and PBTZ169 were less than 5, with values of 4, 4, and 0, respectively. HBA values of hydroxythaxtomin A, lajollamycin B, and PBTZ169 were either equal to or less than 10, with specific values of 7, 10, and 8, respectively. The MW of hydroxythaxtomin A, lajollamycin B, and PBTZ169 were determined as 454.4, 673.8, and 456.5 gm mol^−1^, respectively. Importantly, the slight increase in the MW of lajollamycin B is not expected to substantially affect its transmission and diffusion, given that numerous FDA‐approved drugs exceed the typical molecular weight limit of 500.^[^ ^72^ ^]^ Additionally, the TPSA values of the investigated StreptomeDB compounds were 162.92 and 191.45 Å^2^, respectively, signifying their advantageous properties concerning oral absorption and membrane permeability.^[^ ^73^ ^]^ Besides, hydroxythaxtomin A, lajollamycin B, and PBTZ169 exhibited MLog*P* values close to five, with specific values of 1.54, 4.95, and 3.49, respectively, suggesting their good lipophilicity.

### 3.6 ADMET

To elucidate the behavior of the identified StreptomeDB compounds within the human body, an in‐depth investigation of their pharmacokinetic properties was conducted. ADMET properties of hydroxythaxtomin A (SDB12202), lajollamycin B (SDB9226), and PBTZ169 were predicted utilizing pkCSM online server (**Table** **3**). In line with the absorption (A) data listed in Table 3, hydroxythaxtomin A, lajollamycin B, and PBTZ169 were found to have Caco‐2 permeability values of 0.26, 0.28, and 0.61 cm s^−1^. The Caco‐2 permeability is determined high if its value >0.90 cm s^−1^.^[^ ^74^ ^]^ Hydroxythaxtomin A, lajollamycin B, and PBTZ169 exhibited low skin permeability (log *Kp*) with values of −2.74, −2.72, and −2.72 cm h^−1^, respectively, suggesting there no possible dermatological effect. To gauge drug distribution (D), the permeability of the blood‐brain barrier (BBB) membrane and the CNS was assessed. Hydroxythaxtomin A, lajollamycin B, and PBTZ169 estimated log BB values of −1.09, −1.43, and −0.9, respectively (Table 3). For CNS permeability, hydroxythaxtomin A, lajollamycin B, and PBTZ169 revealed log PS scores of −2.95, −3.51, and −2.1, respectively (Table 3). These absorption findings suggested that the identified StreptomeDB compounds are unlikely to penetrate the BBB or cross the CNS. Focusing on metabolism (M) predictions, hydroxythaxtomin A and lajollamycin B were confirmed as non‐inhibitors of the CYP3A4 enzyme. Conversely, PBTZ169 was found to inhibit CYP3A4, with additional metabolism testing showing that both the identified StreptomeDB compounds and PBTZ169 function as substrates for the CYP3A4 enzyme, as outlined in Table 3. Furthermore, the total clearance property provided insights into the excretion (E) rates, with values of 0.42, 1.36, and 0.02 log ml/min/kg of hydroxythaxtomin A, lajollamycin B, and PBTZ169, respectively. The assessment of the toxicity (T) effect highlighted lajollamycin B as non‐toxic, while hydroxythaxtomin A and PBTZ169 were classified as slightly toxic inhibitors. This study reveals maximum tolerated dose (MTD) values of 0.473, −0.51, and 0.003 mg/kg/day for hydroxythaxtomin A, lajollamycin B, and PBTZ169, respectively, shedding light on their distinct toxicity profiles. All three compounds lack hERG I inhibitory activity, indicating low cardiotoxicity risk, but inhibit hERG II. Moreover, the skin sensitivity test confirmed the absence of adverse reactions, ensuring the safety of contact with these proposed StreptomeDB compounds. Regarding cell wall penetration, potential structural modifications could be utilized to enhance the compounds’ ability to traverse the mycobacterial cell wall. Figure S4, Supporting Information illustrates the strategies for structural modifications of hydroxythaxtomin A and lajollamycin B to facilitate penetration through the mycobacterial cell wall.

**Table 3:** ADMET of hydroxythaxtomin a, lajollamycin B, and PBTZ169.

StreptomeDB Code/Name	Absorption (A)		Distribution (D)		Metabolism (M)		Excretion (E)	Toxicity (T)				
	Caco2 permeability	Skin permeability [log *Kp*]	Blood Brain Barrier (BBB)	CNS permeability	CYP3A4 substrate	CYP3A4 inhibitor	Total Clearance	AMES toxicity	Maximum Tolerated Dose (MTD)	hERG I inhibitor	hERG II inhibitor	skin sensitization
PBTZ169 (Macozinone)	0.61	−2.72	−0.9	−2.1	Yes	Yes	0.02	Yes	0.003	No	Yes	No
(SDB12202) Hydroxythaxtomin A	0.26	−2.74	−1.09	−2.95	Yes	No	0.42	Yes	0.473	No	Yes	No
(SDB9226) Lajollamycin B	0.28	−2.72	−1.43	−3.51	Yes	No	1.36	No	− 0.51	No	Yes	No

Overall, hydroxythaxtomin A and lajollamycin B showed encouraging results, with certain values much surpassing PBTZ169, as detailed in Table 3.

## 4 Conclusion

TB is commonly viewed as one of the most lethal infections in humans, posing a significant global burden in terms of mortality and morbidity. This emphasizes the necessity of tuberculosis treatment through novel anti‐TB agents that can help reduce the worldwide burden of TB. DprE1 is a prospective target of resistant TB, particularly in cases of multidrug‐resistant (MDR) and extensively drug‐resistant (XDR) TB. Recently, PBTZ169 (macozinone, a BTZ derivative) has emerged as a likely irreversible covalent inhibitor of DprE1, especially when combined with additional anti‐TB drugs. The StreptomeDB database is a valuable resource for identifying potential drug candidates derived from Streptomyces species. In the current work, advanced in‐silico techniques were employed to assess 63 nitro‐containing StreptomeDB compounds against DprE1. To pinpoint the most potent DprE1 inhibitors, the StreptomeDB compounds were covalently docked against DprE1. As per the covalent docking scores, the most effective StreptomeDB compounds underwent MDS, accompanied by binding energy predictions via the MM‐GBSA approach. Hydroxythaxtomin A (SDB12202) and lajollamycin B (SDB9226) demonstrated favorable binding affinities with Δ*G*_binding_ values of –51.2 and –50.5 kcal mol^−1^, respectively, compared to PBTZ169 (calc. –49.3 kcal mol^−1^). Relying on post‐dynamics analysis, the examined StreptomeDB compounds revealed significant stability inside the DprE1 binding pocket over 300 ns MDS. Finally, the anticipated physicochemical and ADMET properties suggested that the investigated StreptomeDB compounds possess good oral bioavailability as promising DprE1 inhibitors. The current in‐silico outcomes suggest that hydroxythaxtomin A and lajollamycin B have the potential to serve as effective DprE1 inhibitors, meriting further in‐vivo/in‐vitro investigations.

## Conflict of Interest

The authors declare no conflict of interest.

## Author Contributions

**Doaa G. M. Mahmoud**: formal analysis; investigation; data curation; visualization; writing—original drafts. **Gamal A. H. Mekhemer**: supervision; writing—review and editing. **Mohamed‐Elamir F. Hegazy**: supervision; visualization; writing—review and editing. **Jabir H. Al‐Fahemi**: methodology; resources; writing—review and editing. **Mahmoud A. A. Ibrahim**: conceptualization; methodology; software; resources; project administration; supervision; writing—review and editing. All authors have read and agreed to the published version of the manuscript.

## Supporting information

Supplementary Material

## Data Availability

The data that support the findings of this study are available in the supplementary material of this article.
