# Supplementary material for: In‐Silico Exploration of the StreptomeDB Database for Potential Irreversible DprE1 Inhibitors toward Antitubercular Treatment
Source: ChemistryOpen. 2025 Jul 14;14(11):e202500237. doi: 10.1002/open.202500237 (PMC12598795; doi:10.1002/open.202500237)
Supplement: Supplementary file 1 — Supplementary Material [file OPEN-14-e202500237-s001.pdf]

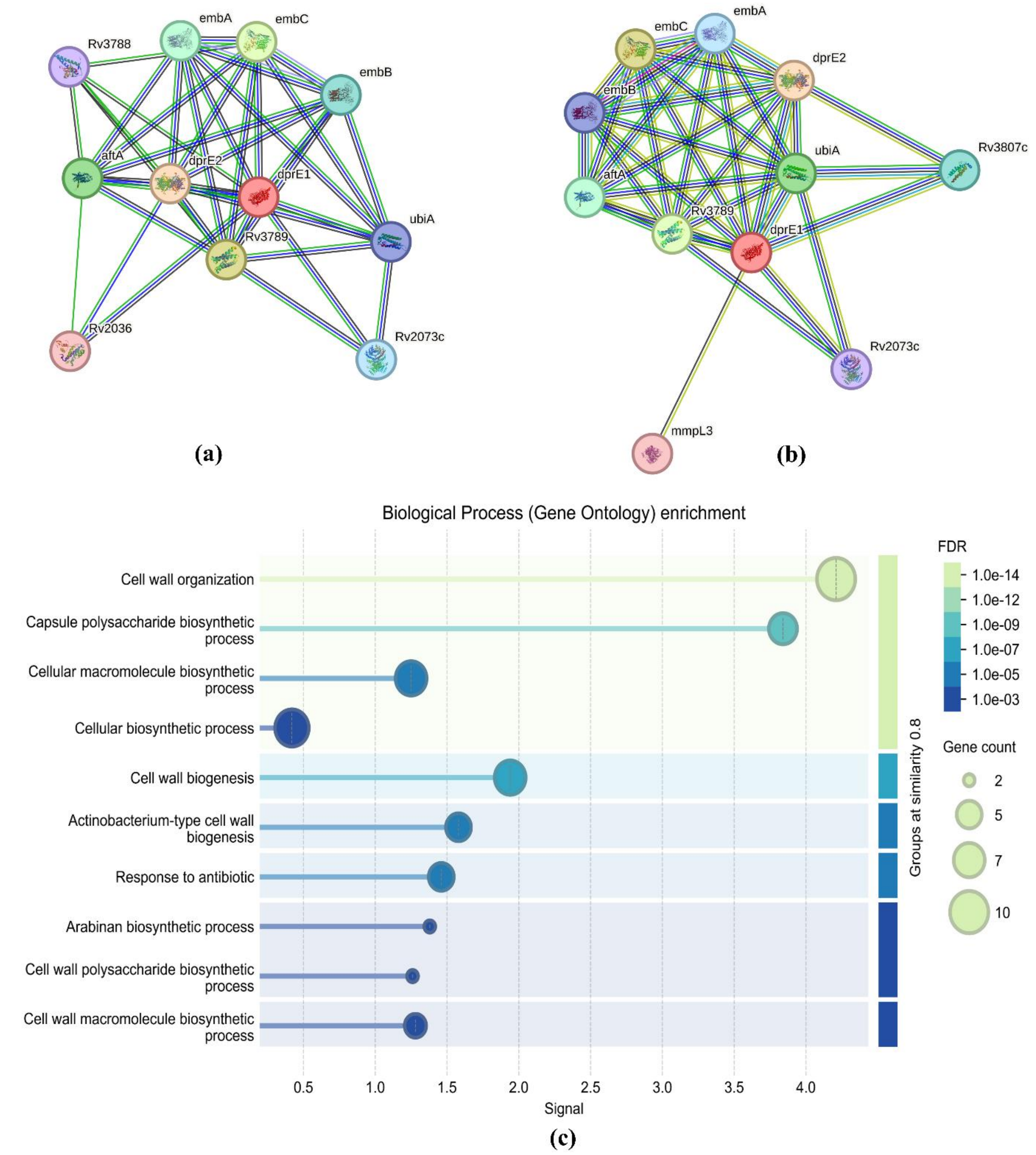

Figure S1. (a) Gene-gene interaction network analysis, (b) protein-protein interaction network analysis, and (c) Gene Ontology analysis of DprE1-associated proteins.

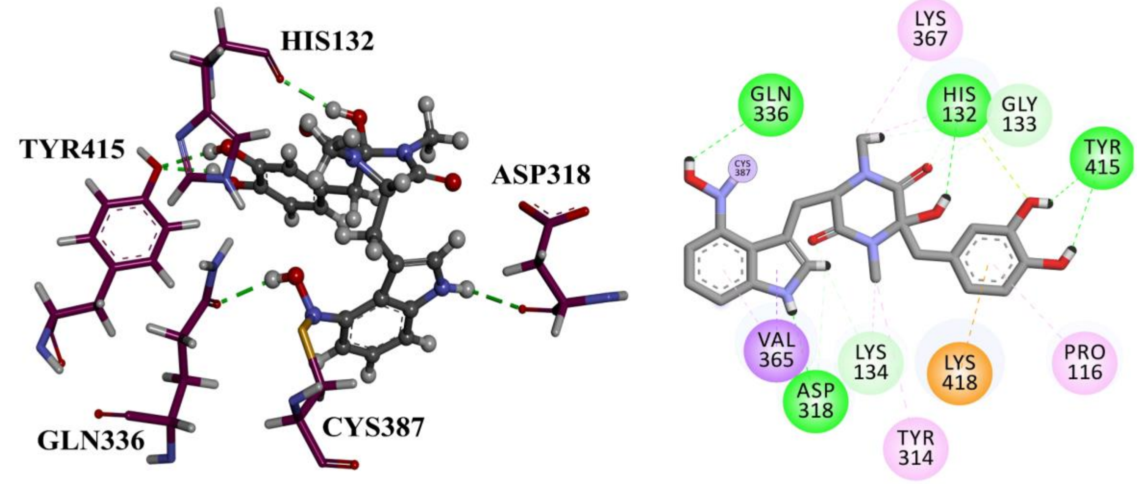

SDB12202

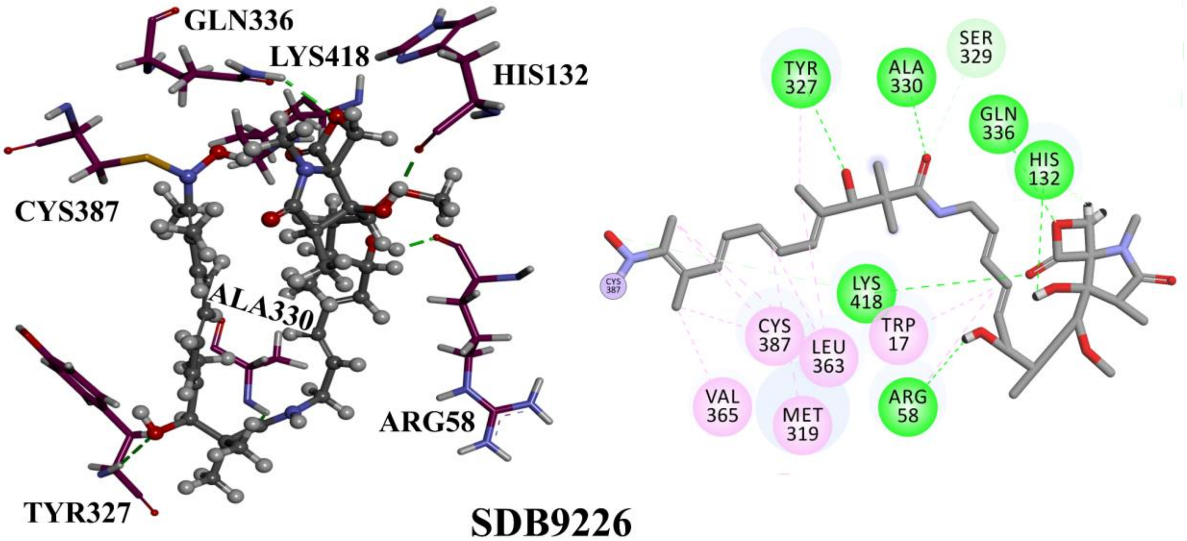

SDB9226

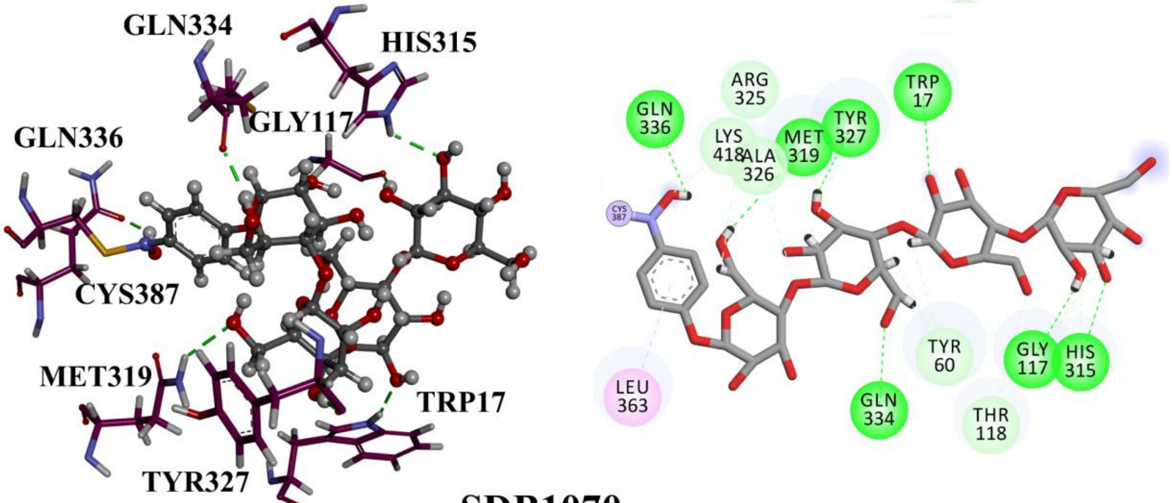

SDB1070

Figure S2. 3D and 2D representations of the binding modes of the most potent StreptomeDB compounds in complex with DprE1.

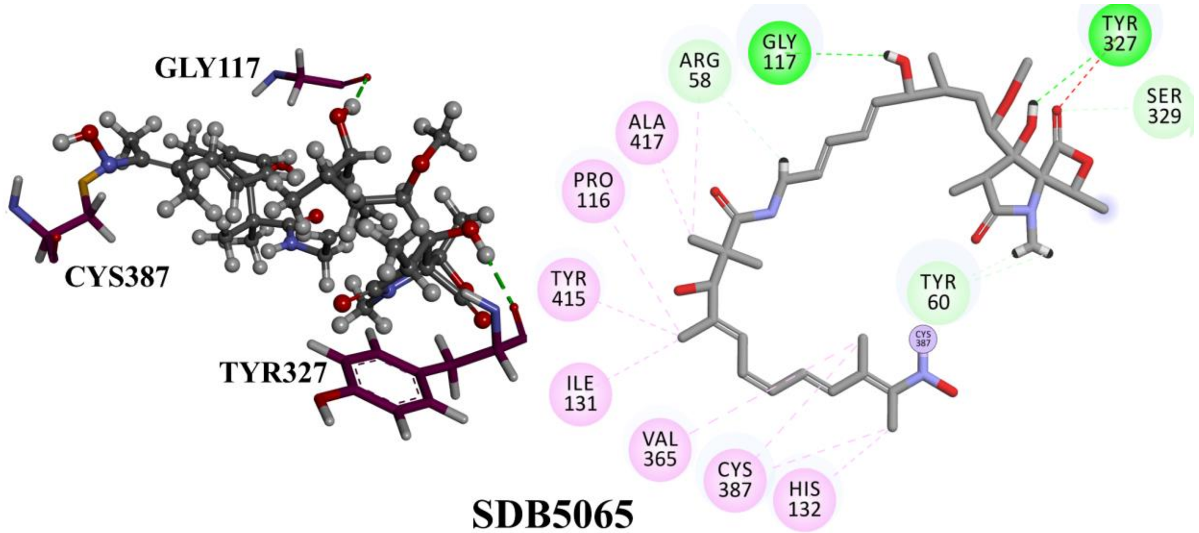

SDB5065

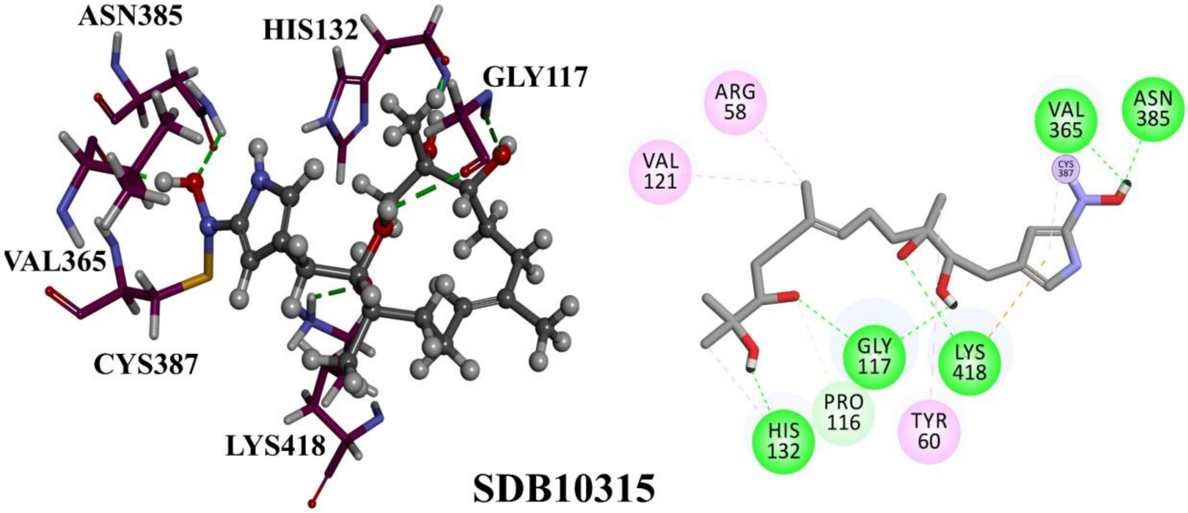

SDB10315

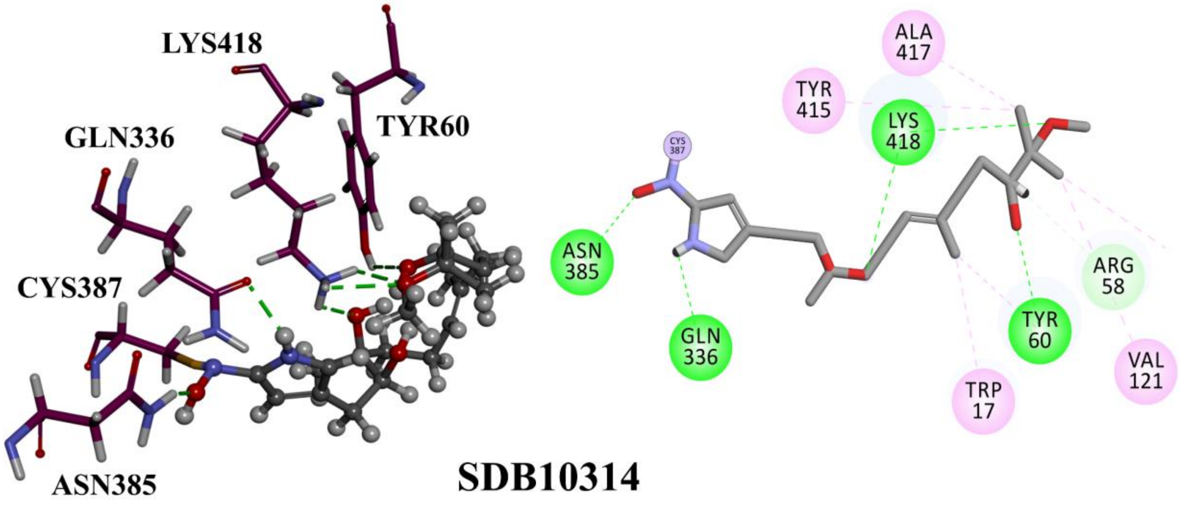

SDB10314

Figure S2. *Continued*.

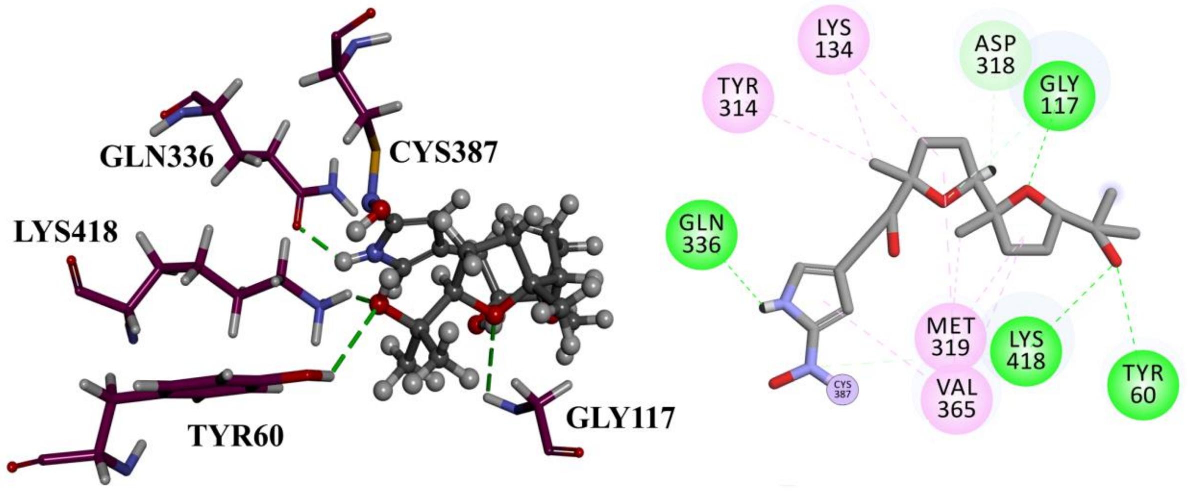

SDB9277

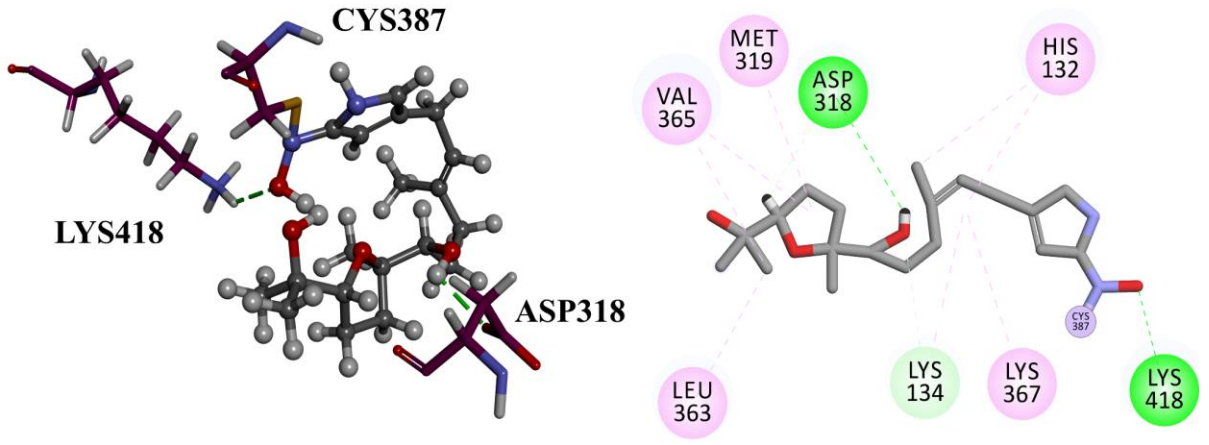

SDB9276

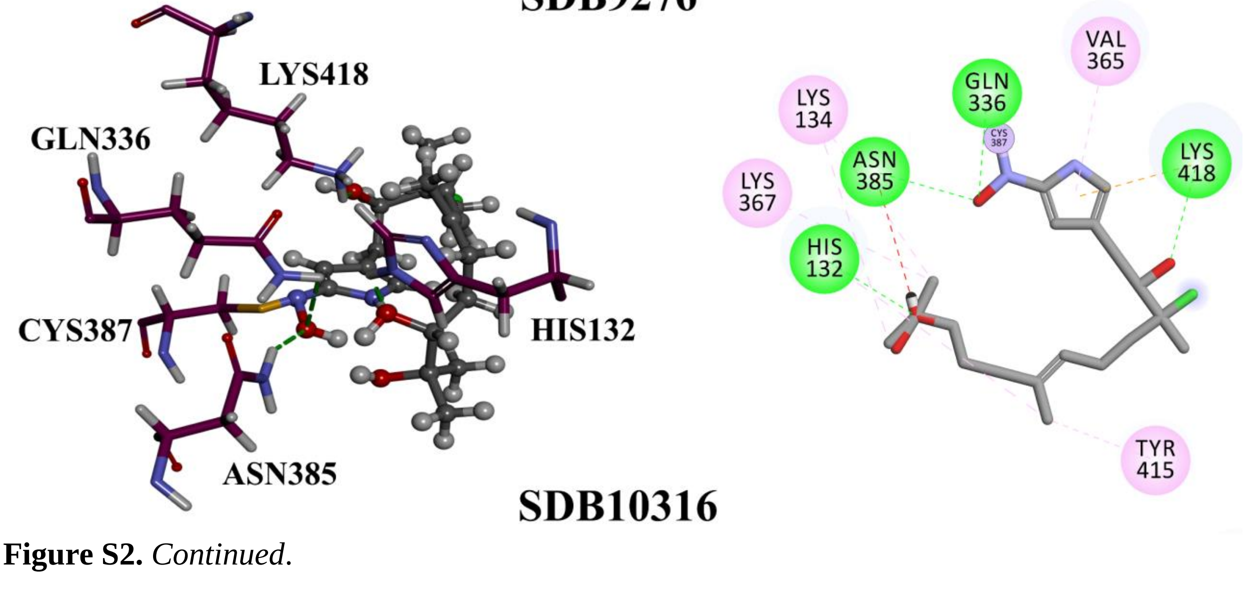

SDB10316

Figure S2. *Continued*.

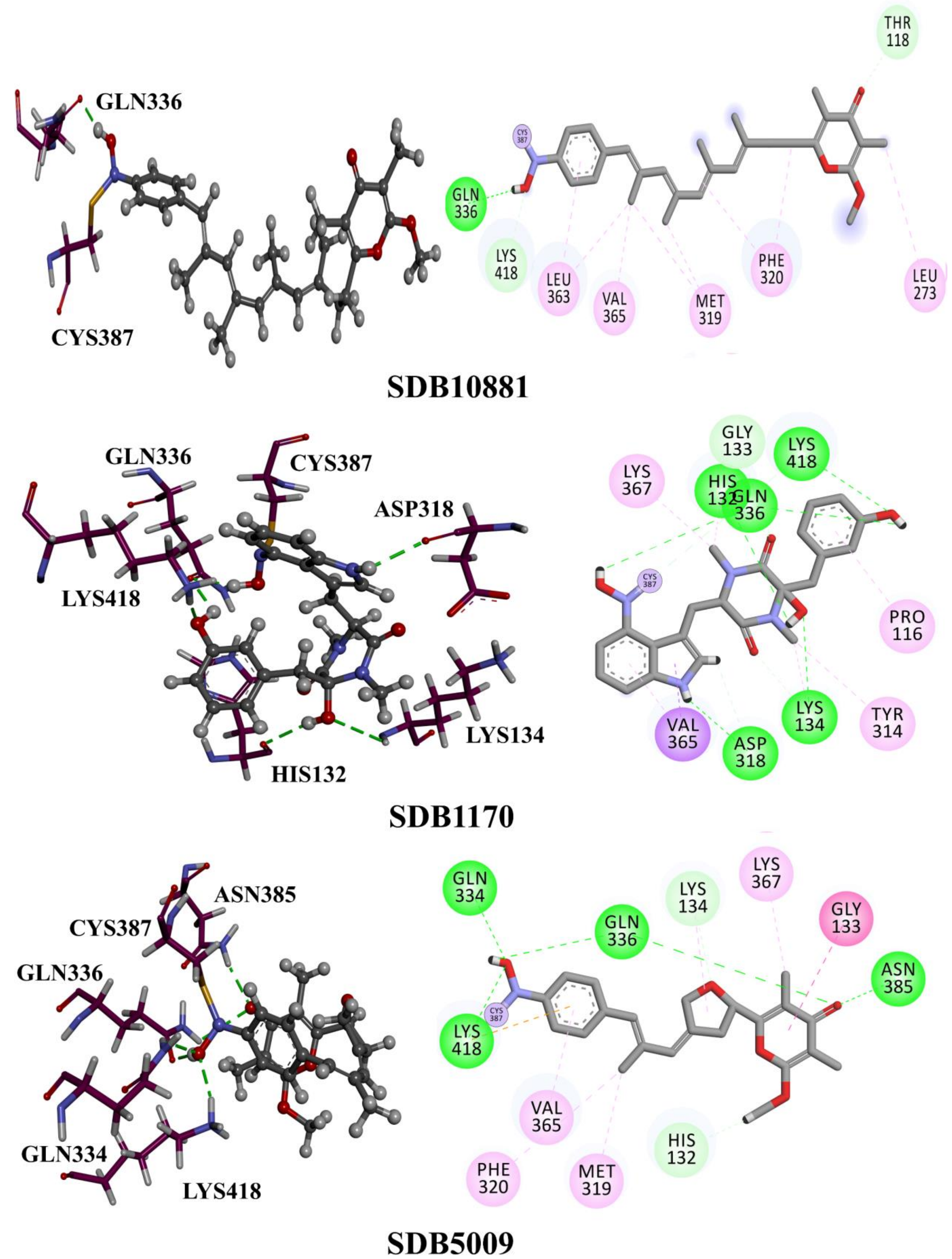

Figure S2. *Continued*.

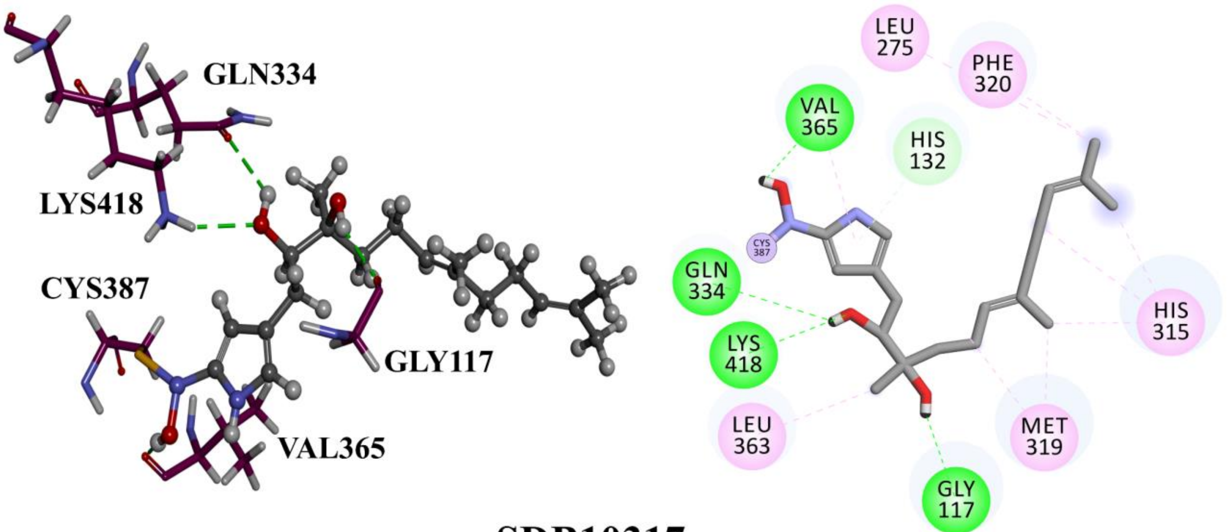

SDB10317

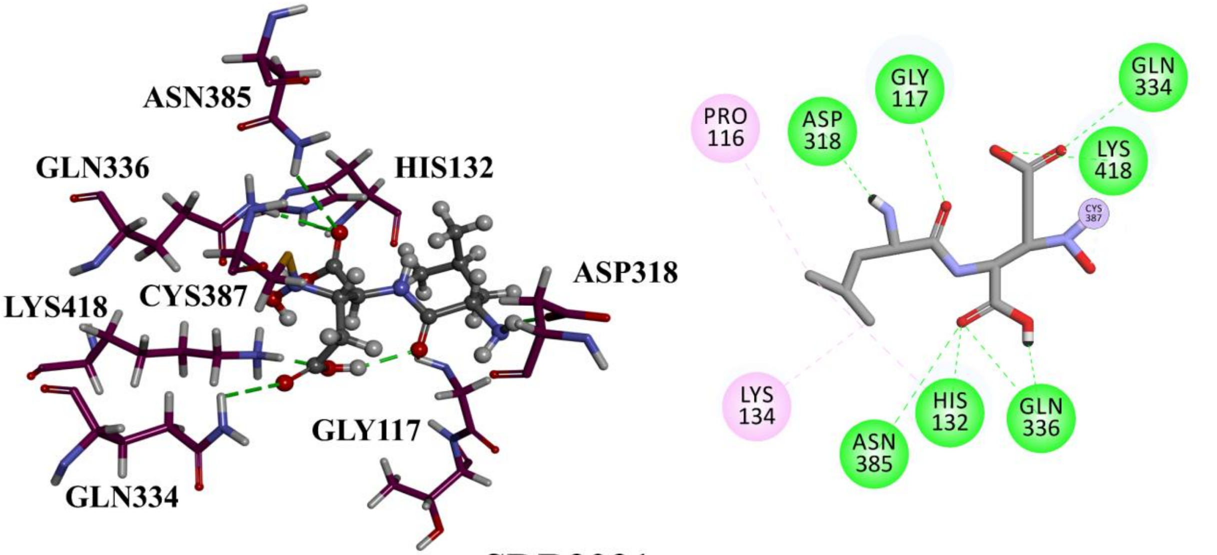

SDB3031

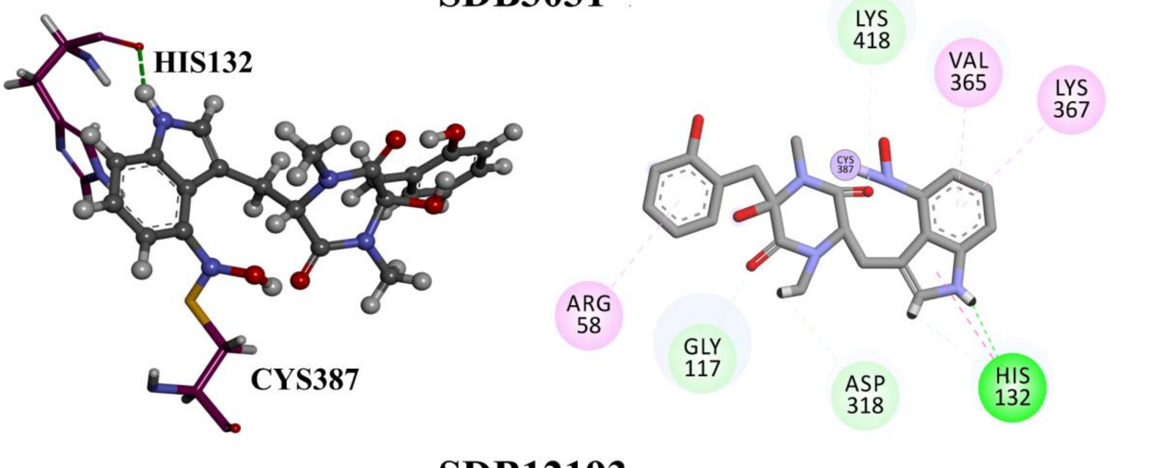

SDB12193

Figure S2. *Continued*.

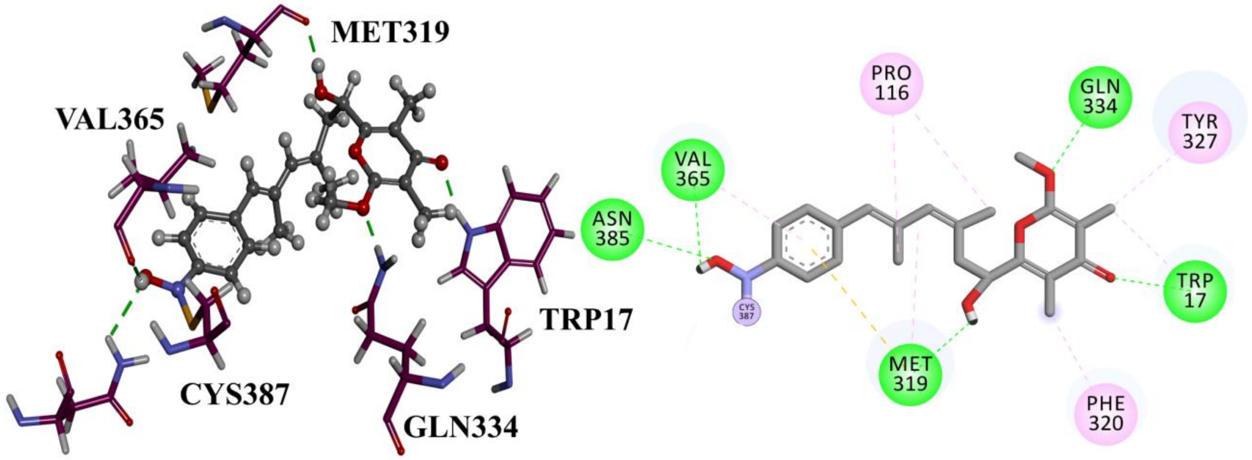

SDB9443

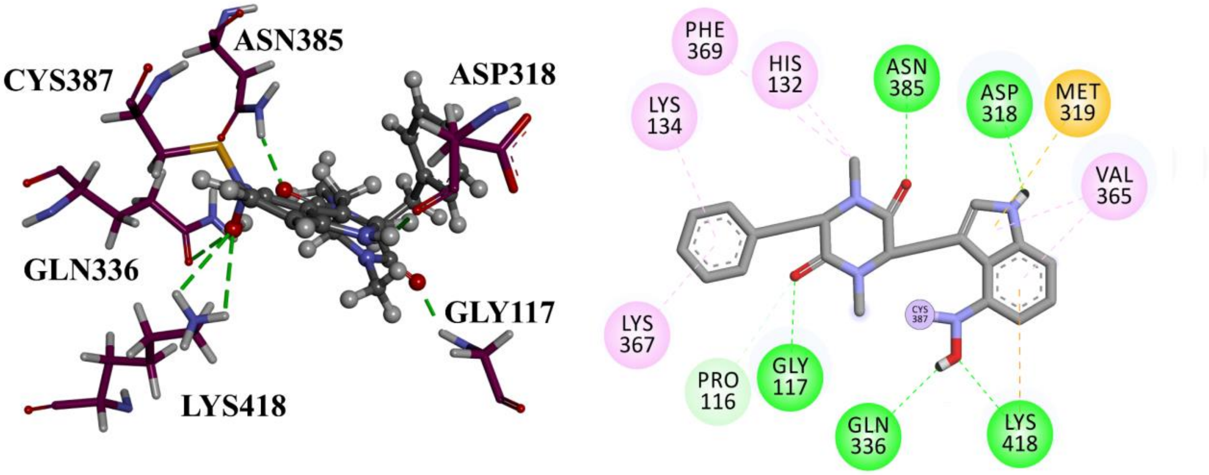

SDB12195

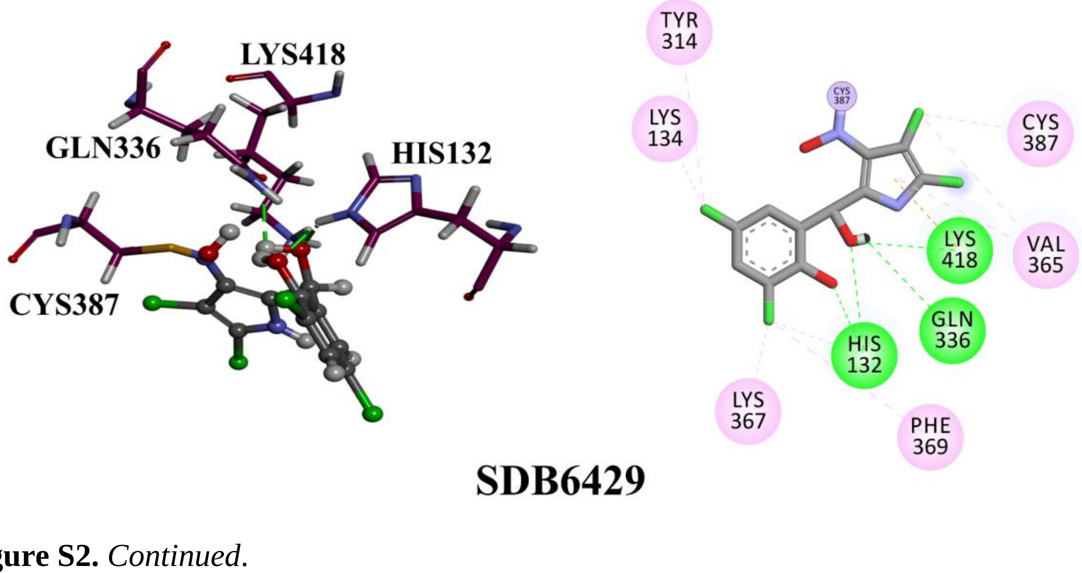

SDB6429

Figure S2. *Continued*.

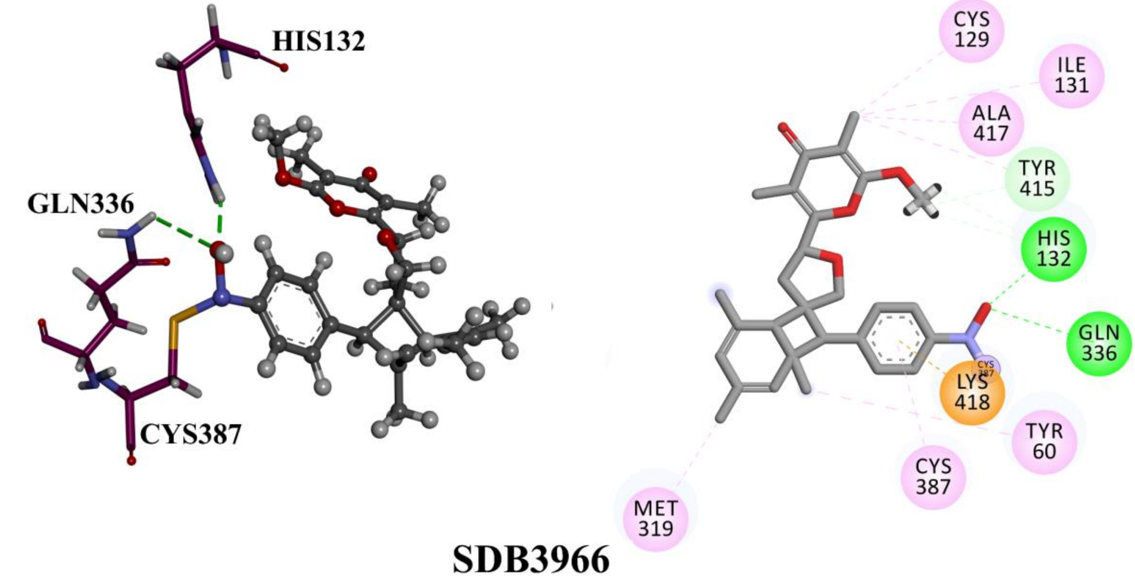

SDB3966

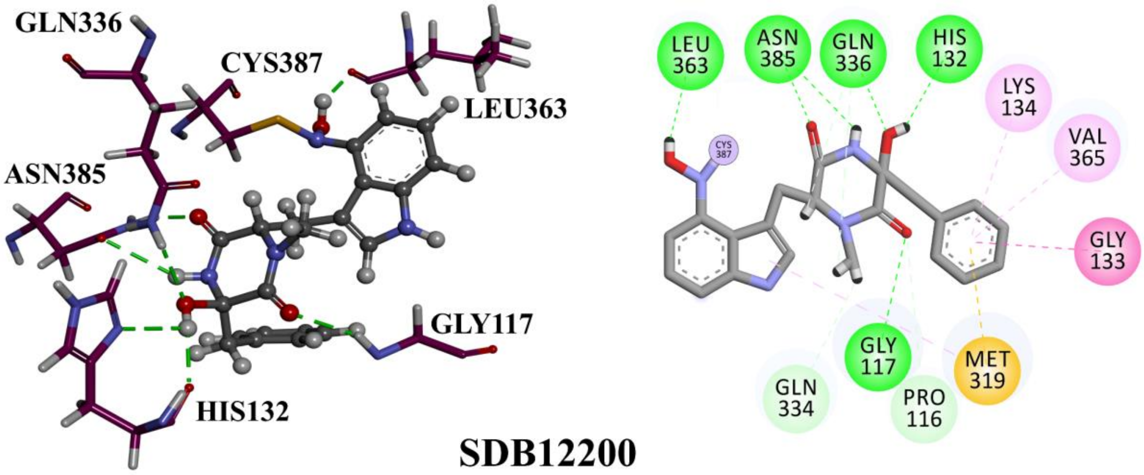

SDB12200

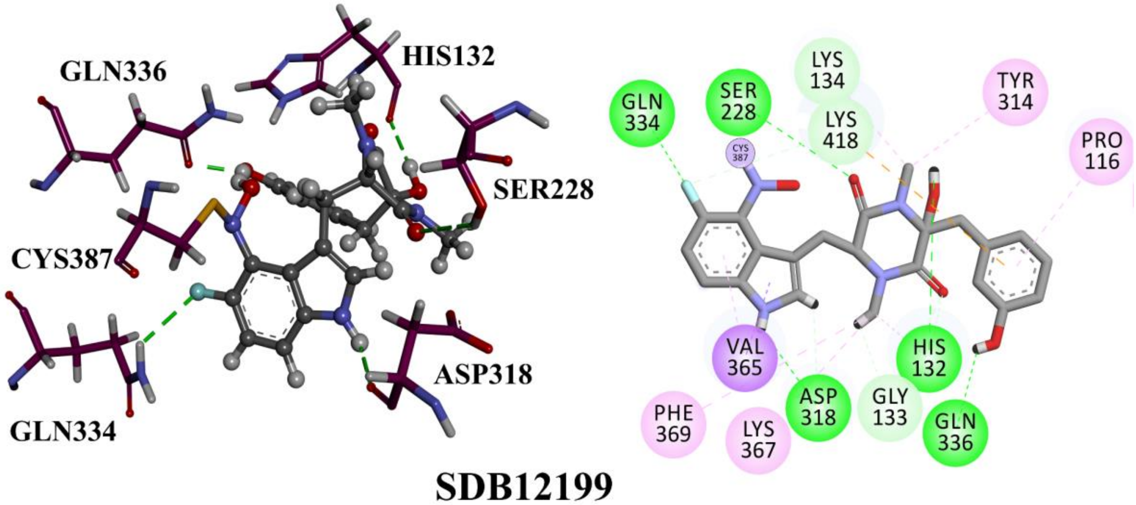

SDB12199

Figure S2. *Continued*.

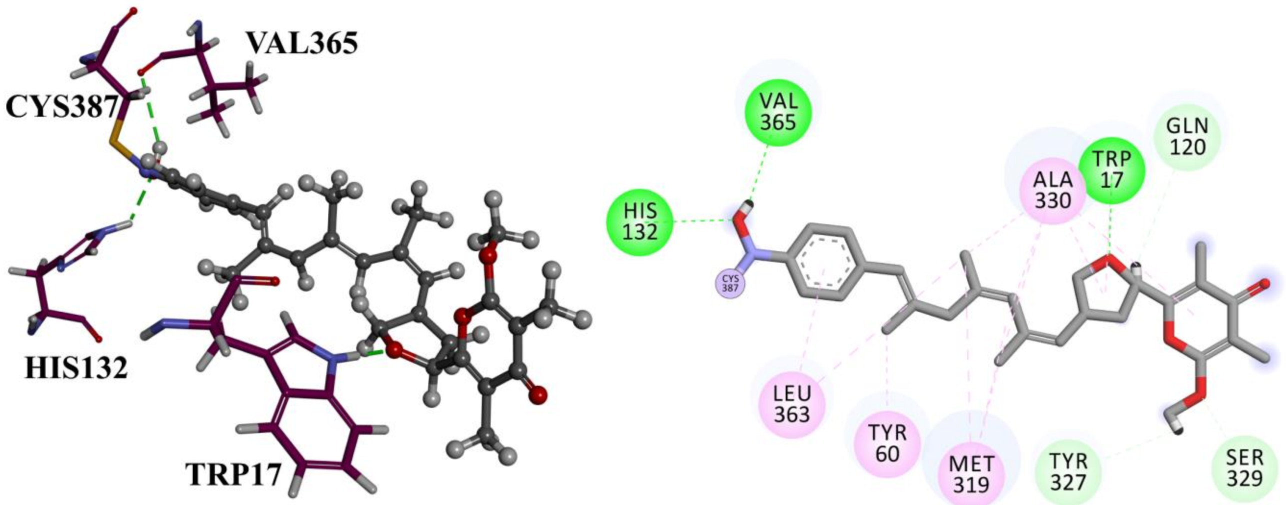

SDB5006

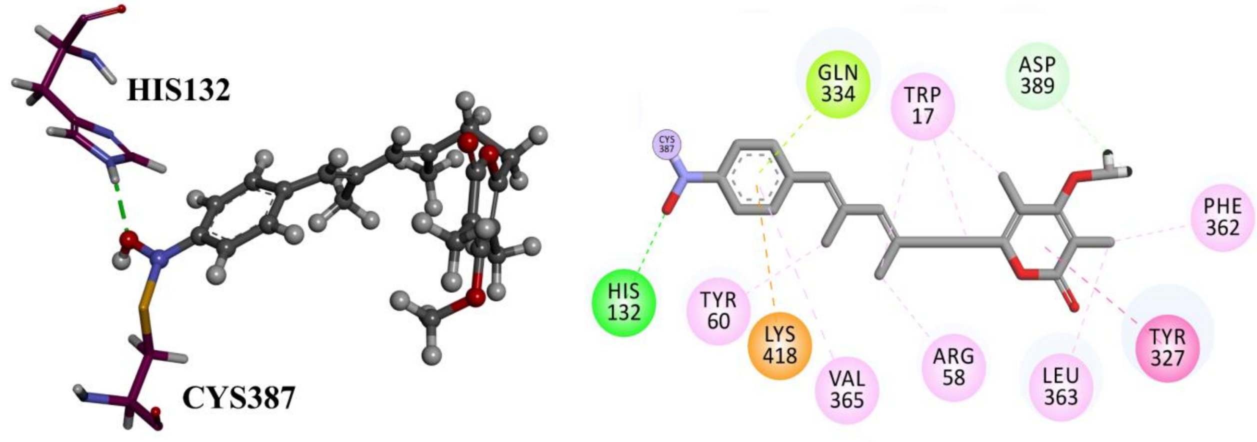

SDB10308

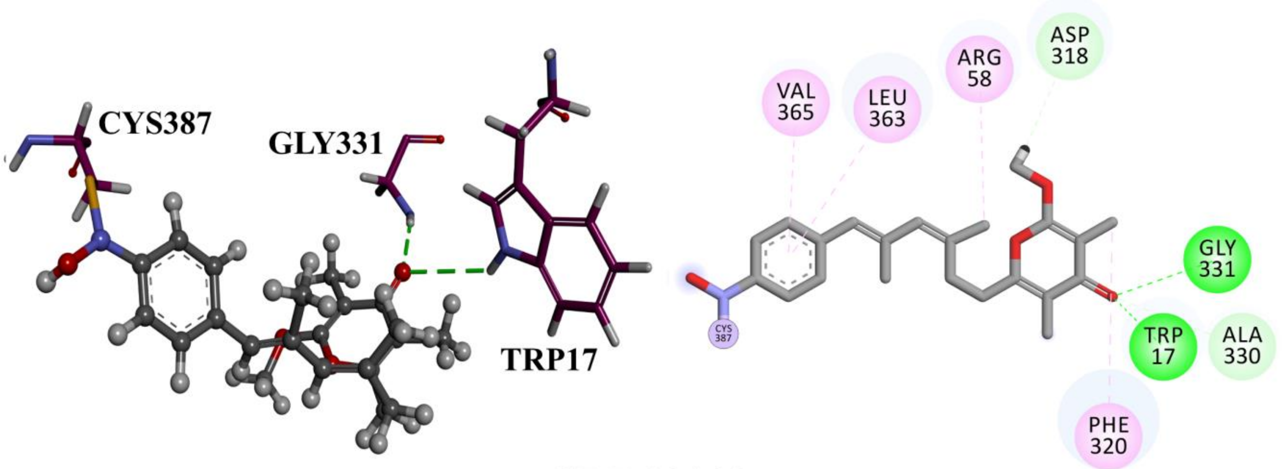

SDB10100

Figure S2. *Continued*.

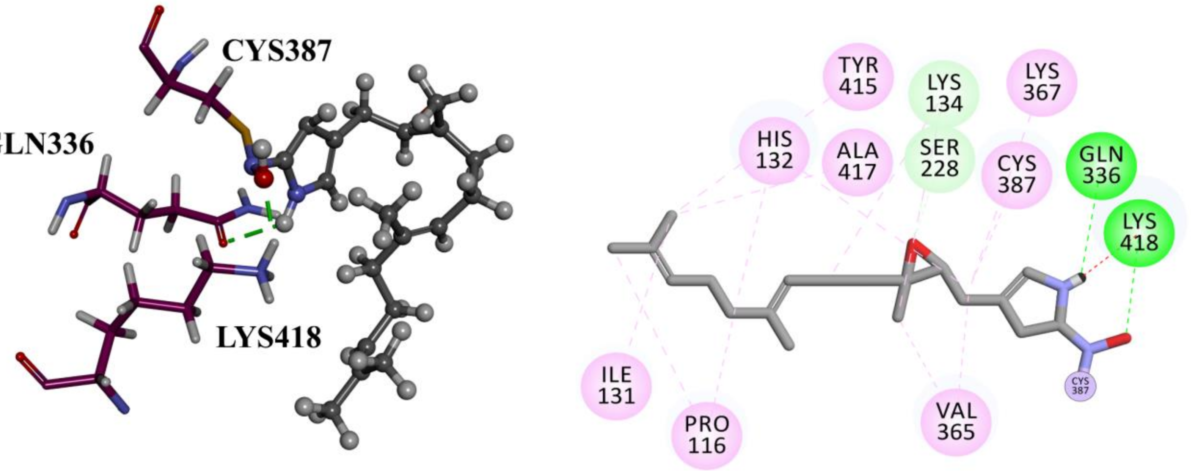

SDB10318

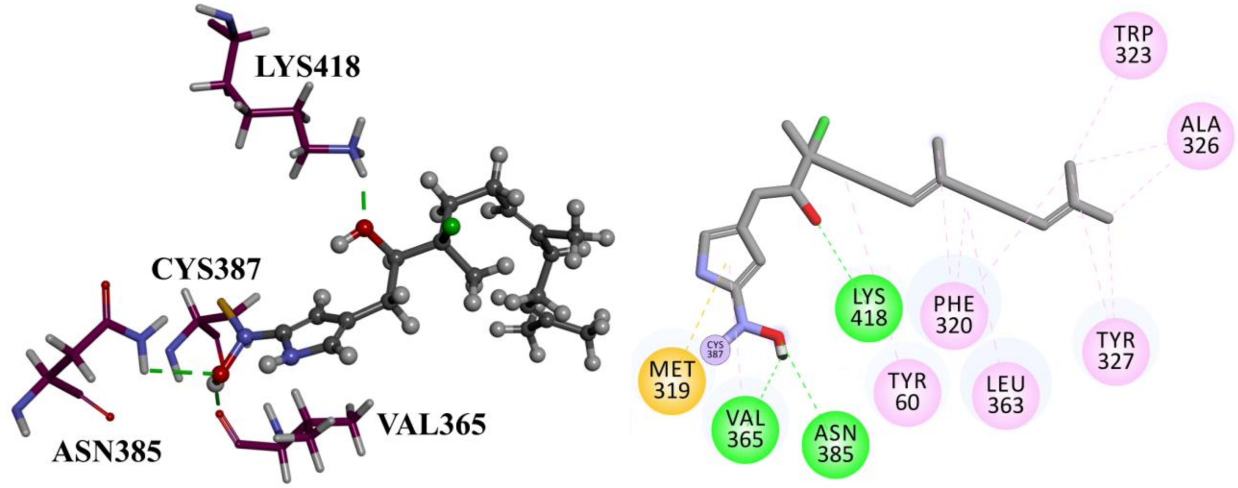

SDB10319

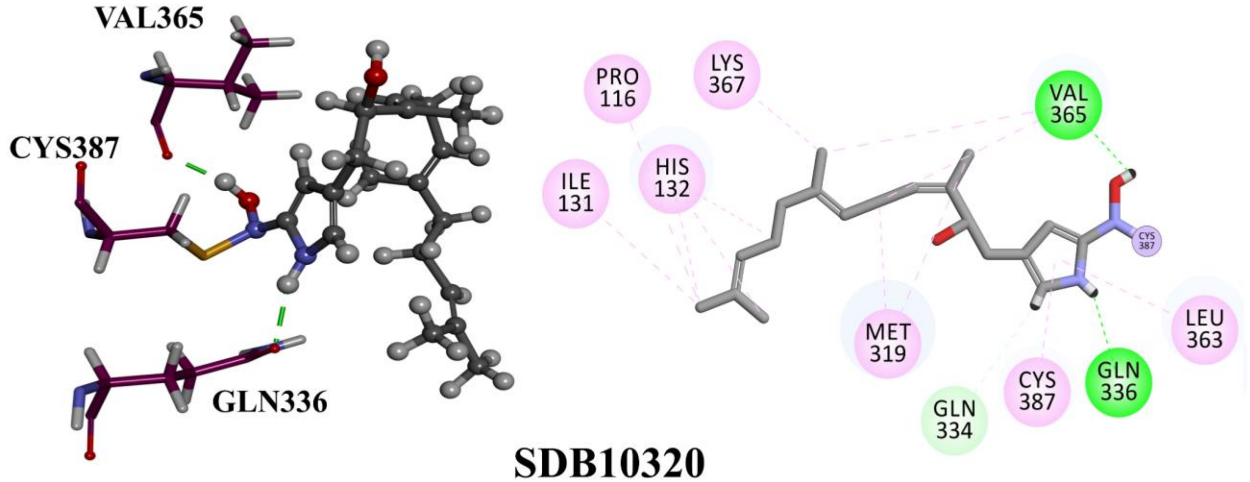

SDB10320

Figure S2. *Continued*.

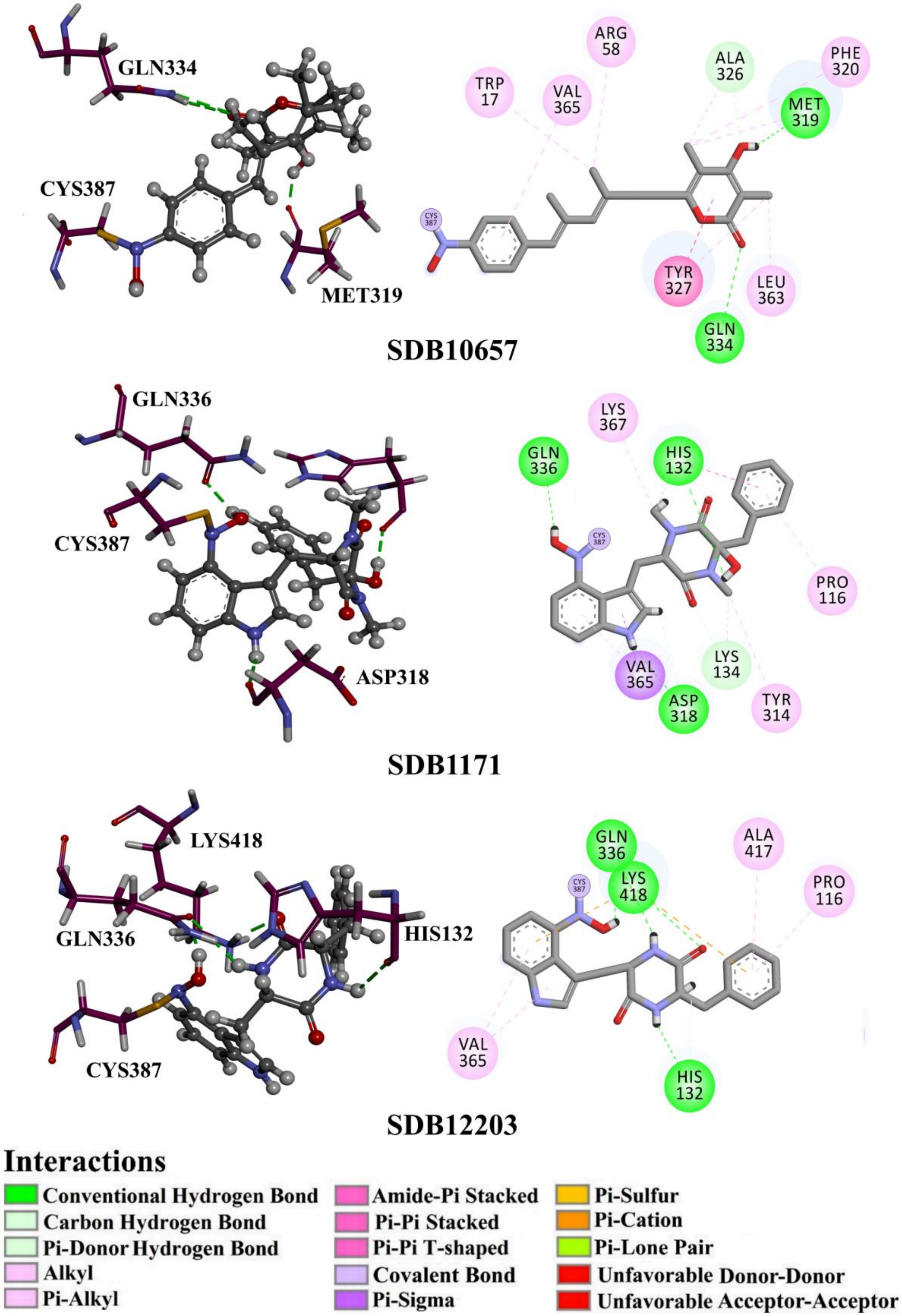

Figure S2. *Continued*.

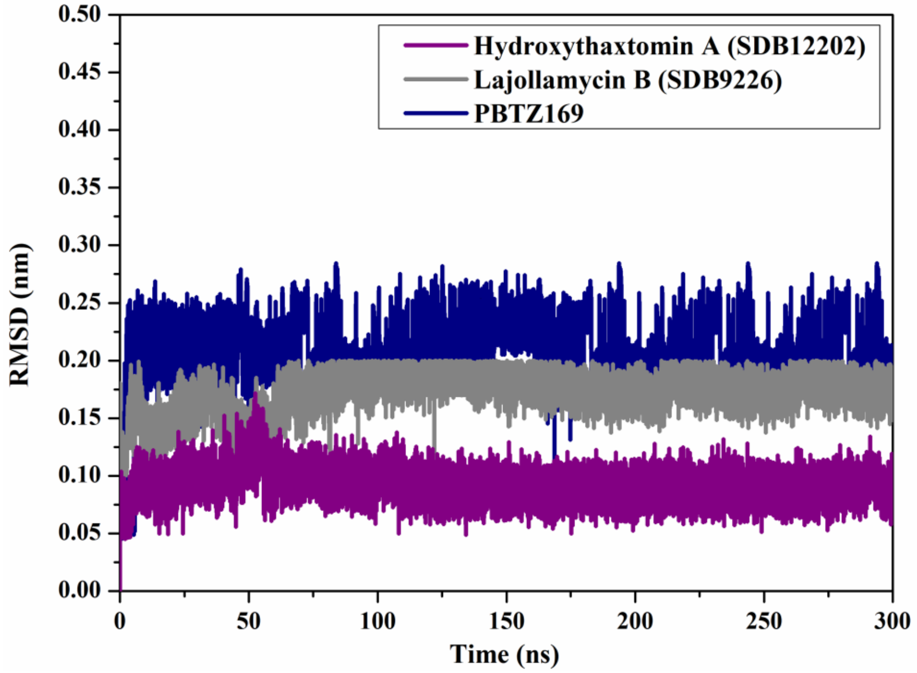

Figure S3. Relative RMSD plots of hydroxythaxtomin A (purple), lajollamycin B (grey), and PBTZ169 (dark blue) against the DprE1 a 300 ns MDS period.

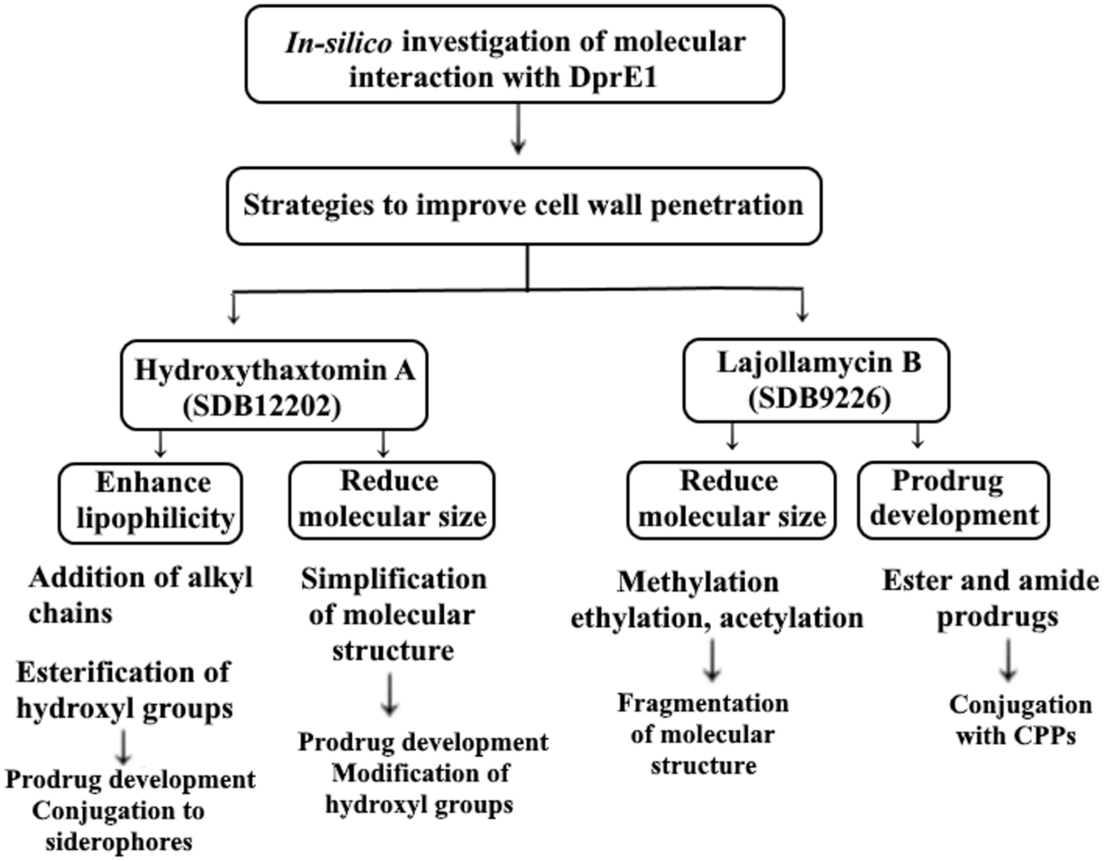

Figure S4. Strategies for structural modification of hydroxythaxtomin A and lajollamycin B to facilitate penetration through the mycobacterial cell wall.

Table S1. Estimated covalent docking scores (in kcal/mol) of PBTZ169 and 63 StreptomeDB compounds against DprE1.

No.	StreptomeDB Code	Covalent Docking Score (kcal/mol)	No.	StreptomeDB Code	Covalent Docking Score (kcal/mol)
	PBTZ169	–7.8	32	SDB12196	–7.2
1	SDB12202	–14.2	33	SDB10882	–7.1
2	SDB9226	–13.9	34	SDB3055	–6.9
3	SDB1070	–13.8	35	SDB13120	–6.8
4	SDB5065	–13.7	36	SDB15	–6.7
5	SDB10315	–12.6	37	SDB6430	–6.6
6	SDB10314	–11.0	38	SDB3572	–6.5
7	SDB9277	–10.7	39	SDB9101	–6.5
8	SDB9276	–10.5	40	SDB5027	–6.3
9	SDB10316	–10.4	41	SDB12194	–6.1
10	SDB10881	–10.3	42	SDB3020	–6.0
11	SDB1170	–9.8	43	SDB12580	–5.4
12	SDB5009	–9.7	44	SDB11296	–5.4
13	SDB10317	–9.2	45	SDB3836	–5.2
14	SDB3031	–9.0	46	SDB3791	–5.0
15	SDB12193	–8.9	47	SDB4977	–5.0
16	SDB9443	–8.9	48	SDB12197	–4.8
17	SDB12195	–8.7	49	SDB13101	–4.8
18	SDB6429	–8.6	50	SDB5099	–4.4
19	SDB3966	–8.5	51	SDB12198	–4.3
20	SDB12200	–8.5	52	SDB9934	–4.2
21	SDB12199	–8.3	53	SDB2891	–3.9
22	SDB5006	–8.3	54	SDB10999	–3.8
23	SDB10308	–8.3	55	SDB813	–3.6
24	SDB10100	–8.3	56	SDB9800	–3.2
25	SDB10318	–8.1	57	SDB3124	–3.1
26	SDB10319	–8.1	58	SDB12238	–2.9
27	SDB10320	–8.0	59	SDB4808	–2.8
28	SDB10657	–8.0	60	SDB3056	–2.8
29	SDB1171	–7.9	61	SDB2874	–2.8
30	SDB12203	–7.9	62	SDB12256	–2.7
31	SDB13121	–7.7	63	SDB3698	–1.5

Table S2. Calculated covalent docking scores and MM-GBSA binding energies (in kcal/mol) over 5 ns MDS of PBTZ169 and the top 30 StreptomeDB compounds against DprE1 [a].

No.	StreptomeDB Code	Covalent Docking Score (kcal/mol)	MM–GBSA Binding Energy (kcal/mol)
	PBTZ169	–7.8	–37.9
1	SDB9226	–13.9	–50.3
2	SDB5065	–13.7	–53.3
3	SDB9276	–10.5	–46.5
4	SDB1171	–7.9	–46.3
5	SDB10315	–12.6	–45.5
6	SDB10316	–10.4	–44.8
7	SDB12193	–8.9	–44.2
8	SDB1170	–9.8	–43.6
9	SDB12203	–7.9	–42.4
10	SDB10318	–8.1	–41.4
11	SDB9277	–10.7	–39.7
12	SDB10319	–8.1	–38.3
13	SDB12202	–14.2	–38.2
14	SDB6429	–8.6	–38.0
15	SDB12195	–8.7	–37.6
16	SDB10320	–8.0	–35.9
17	SDB5006	–8.3	–35.2
18	SDB10314	–11.0	–34.7
19	SDB5009	–9.7	–34.6
20	SDB12200	–8.5	–33.7
21	SDB10100	–8.3	–33.6
22	SDB12199	–8.3	–33.0
23	SDB9443	–8.9	–32.7
24	SDB3031	–9.0	–32.0
25	SDB10317	–9.2	–30.6
26	SDB1070	–13.8	–26.7
27	SDB10308	–8.3	–24.5
28	SDB10881	–10.3	–23.8
29	SDB10657	–8.0	–23.6
30	SDB3966	–8.5	–10.5

[a] Data ranked based on the MM-GBSA binding energy over 5 ns MDS.

Table S3. Estimated covalent docking scores and MM-GBSA binding energies (in kcal/mol) over 5 and 25 ns MDS of PBTZ169 and the top 14 StreptomeDB compounds against DprE1 [a].

No.	StreptomeDB Code	Covalent Docking Score (kcal/mol)	MM–GBSA Binding Energy (kcal/mol)	
			5 ns	25 ns
	PBTZ169	–7.8	–37.9	–42.9
1	SDB9226	–13.9	–50.3	–50.8
2	SDB5065	–13.7	–53.3	–49.4
3	SDB1171	–7.9	–46.3	–48.1
4	SDB12193	–8.9	–44.2	–45.8
5	SDB12202	–14.2	–38.2	–44.4
6	SDB1170	–9.8	–43.6	–43.5
7	SDB12203	–7.9	–42.4	–43.4
8	SDB10315	–12.6	–45.5	–43.2
9	SDB9276	–10.5	–46.5	–40.2
10	SDB10316	–10.4	–44.8	–40.1
11	SDB10318	–8.1	–41.4	–38.8
12	SDB6429	–8.6	–38.0	–38.2
13	SDB9277	–10.7	–39.7	–37.8
14	SDB10319	–8.1	–38.3	–35.3

[a] Data ranked based on the MM-GBSA binding energy during 25 ns MDS.

Table S4. Calculated covalent docking scores and MM-GBSA binding energies (in kcal/mol) over 5, 25, and 50 ns MDS of PBTZ169 and the top 8 StreptomeDB compounds with DprE1[a].

No.	StreptomeDB Code	Covalent Docking Score (kcal/mol)	MM–GBSA Binding Energy (kcal/mol)		
			5 ns	25 ns	50 ns
	PBTZ169	–7.8	–37.9	–42.9	–46.9
1	SDB9226	–13.9	–50.3	–50.8	–50.7
2	SDB5065	–13.7	–53.3	–49.4	–47.8
3	SDB1171	–7.9	–46.3	–48.1	–46.8
4	SDB12202	–14.2	–38.2	–44.4	–45.8
5	SDB12193	–8.9	–44.2	–45.8	–45.1
6	SDB10315	–12.6	–45.5	–43.2	–43.6
7	SDB1170	–9.8	–43.6	–43.5	–43.4
8	SDB12203	–7.9	–42.4	–43.4	–40.6

[a] Data ranked according to the MM-GBSA binding energy over the 50 ns MDS.

Table S5. Calculated covalent docking scores and MM-GBSA binding energies (in kcal/mol) over 5, 25, 50, and 100 ns MDS of PBTZ169 and the top 5 StreptomeDB compounds with DprE1 [a].

No.	StreptomeDB Code	Covalent Docking Score (kcal/mol)	MM–GBSA Binding Energy (kcal/mol)			
			5 ns	25 ns	50 ns	100 ns
	PBTZ169	–7.8	–37.9	–42.9	–46.9	–49.8
1	SDB9226	–13.9	–50.3	–50.8	–50.7	–50.2
2	SDB12202	–14.2	–38.2	–44.4	–45.8	–48.3
3	SDB12193	–8.9	–44.2	–45.8	–45.1	–44.6
4	SDB5065	–13.7	–53.3	–49.4	–47.8	–43.8
5	SDB1171	–7.9	–46.3	–48.1	–46.8	–43.0

[a] Data ranked based on the MM-GBSA binding energy over the 100 ns MDS.
